# Transcriptomic profiling of purified patient-derived dopamine neurons identifies convergent perturbations and therapeutics for Parkinson’s disease

**DOI:** 10.1093/hmg/ddw412

**Published:** 2017-01-17

**Authors:** Cynthia Sandor, Paul Robertson, Charmaine Lang, Andreas Heger, Heather Booth, Jane Vowles, Lorna Witty, Rory Bowden, Michele Hu, Sally A. Cowley, Richard Wade-Martins, Caleb Webber

**Affiliations:** 1 Oxford Parkinson's Disease Centre, Department of Physiology, Anatomy and Genetics, Le Gros Clark Building, University of Oxford, Oxford, UK; 2 MRC Functional Genomics Unit, Department of Physiology, Anatomy and Genetics, University of Oxford, Oxford, UK; 3 MRC Computational Genomics Analysis and Training Program, MRC Functional Genomics Unit, Department of Physiology, Anatomy and Genetics, University of Oxford, Oxford, UK; 4 James Martin Stem Cell Facility, Sir William Dunn School of Pathology, University of Oxford, Oxford, UK; 5 Wellcome Trust Centre for Human Genetics, University of Oxford, Oxford, UK; 6 Nuffield Department of Clinical Neurosciences, University of Oxford, Oxford, UK

## Abstract

While induced pluripotent stem cell (iPSC) technologies enable the study of inaccessible patient cell types, cellular heterogeneity can confound the comparison of gene expression profiles between iPSC-derived cell lines. Here, we purified iPSC-derived human dopaminergic neurons (DaNs) using the intracellular marker, tyrosine hydroxylase. Once purified, the transcriptomic profiles of iPSC-derived DaNs appear remarkably similar to profiles obtained from mature post-mortem DaNs. Comparison of the profiles of purified iPSC-derived DaNs derived from Parkinson’s disease (PD) patients carrying *LRRK2 G2019S* variants to controls identified significant functional convergence amongst differentially-expressed (DE) genes. The PD *LRRK2-G2019S* associated profile was positively matched with expression changes induced by the Parkinsonian neurotoxin rotenone and opposed by those induced by clioquinol, a compound with demonstrated therapeutic efficacy in multiple PD models. No functional convergence amongst DE genes was observed following a similar comparison using non-purified iPSC-derived DaN-containing populations, with cellular heterogeneity appearing a greater confound than genotypic background.

## Introduction

Parkinson’s disease (PD) is the most common neurodegenerative movement disorder worldwide, affecting 1% of the population over 65 years, rising to 5% over the age of 85 (1). PD is characterized clinically by motor manifestations, which have largely been attributed to the preferential loss of dopaminergic neurons (DaNs) from the substantia nigra pars compacta, a specific sub-population of midbrain dopaminergic neurons (2). While the majority of PD cases are sporadic, around 10% of patients present with monogenic forms of the disease (3). A common missense mutation, *G2019S*, in the leucine-rich repeat kinase 2 gene (*LRRK2-G2019S*) is of particular interest, as 8% of familial forms and up to 2% of sporadic forms of PD are attributed to this mutation (4–6). *LRKK2-G2019S* mutations predispose towards an autosomal dominant, late-onset familial PD, whose clinical and pathological features are indistinguishable from the common sporadic form of PD, indicating potential overlapping pathways across both familial and sporadic forms (7,8). Which molecular pathway perturbations underlie DaNs cell death in *LRKK2-G2019S* PD patients are currently unclear.

Our poor understanding of the pathogenic mechanisms that lead to PD are in part due to the inaccessibility of the human brain and a lack of appropriate models of the disease (9,10). Most of our current knowledge of the cellular phenotypes involved in PD are derived from end-stage post-mortem brain tissue or rodent models, which either may not allow the study of early stage pathophysiology, may not accurately represent how the disease develops, or fail to recapitulate the pathology of human PD (11–13). In particular, the inability to isolate human DaNs to study their heightened susceptibility to cell death in PD has hampered the study of disease mechanisms (14). Recent advances in induced pluripotent stem cell (iPSC) technology offer the opportunity to reprogram human somatic cells into pluripotent stem cells, which can then be differentiated into disease-specific cell types of interest (15). Deriving these cells from a donor whose genome harbours disease-predisposing alleles provides a model in which to study the contribution of these alleles to disease in hitherto-inaccessible human cell types (16). The differentiation of iPSCs into functional midbrain DaNs provides a powerful tool to study the particular genetic contribution of the *LRKK2-G2019S* mutation to PD in a highly relevant model.

Differentiating iPSCs into midbrain DaNs results in a mixed population comprising a high percentage of DaNs, but also proliferating neural progenitor cells (NPCs) or cells of differing neuronal maturity (17). Therefore, in order to study the specific sensitivity of DaNs in *LRKK2-G2019S* PD it would be crucial to separate this specific subset of cells from the other heterogeneous cell types post-differentiation. The presence of multiple cell types within a culture confounds experimental approaches such as transcriptomics to study DaNs as one is unable to deconvolute the contributions of different cell types within the combined RNA profile. Previous attempts to yield a pure population of cells have used markers for DaN progenitor cells or neurons by fluorescent activated cell sorting (FACS) to enrich for a DaN progenitor/neuronal population. Although these methods increase enrichment, they lack an accurate identification and isolation of DaNs specifically (17–19) and remaining cellular heterogeneity may confound transcriptomic analyses.

To enable transcriptomic analysis of DaNs, we developed an approach to obtain purified populations of DaNs by identifying and isolating DaNs within differentiated iPSC populations by FACS, using a live/dead stain followed by staining for the DaN marker tyrosine hydroxylase (TH). We show that this results in a significantly increased purification required for transcriptomic comparisons. Using lines derived from three controls and three PD patients carrying *LRRK2-G2019S* variants, we demonstrate that upon purification the transcriptome of this purified DaNs model closely matches that obtained from mature post-mortem LCM-captured DaNs, and reveals a functionally-coherent set of genes differentially expressed between the case and control lines. The perturbation in gene expression is significantly similar to that induced by the pesticide rotenone, an environmental cause of PD, and is opposed by clioquinol, a compound shown to have beneficial effects in multiple PD models. However, these results are not observable in a cellularly heterogeneous PD *LRRK2-G2019S* iPSC-derived neuronal model.

## Results

### Purification of iPSC dopaminergic neurons by flow cytometry

Induced pluripotent stem cell (iPSC) lines were derived from three PD patients carrying a *LRRK2-G2019S* heterozygous mutation, and three healthy control individuals (Materials and Methods), from the Oxford Parkinson’s Disease Centre (OPDC) Discovery Cohort (Table 1) (Materials and Methods, Supplementary Material, Fig. S1, Notes S1 and S2). These lines were then differentiated into midbrain dopamine neurons, as previously described by Kriks *et al.* (20), with minor modifications (Materials and Methods).

**Table 1.** Experimental design controls and patients lines used in this study from Oxford Parkinson's Disease Centre (ODPC) Discovery Cohort

Donor ID	Study ID	iPSc Clone	TH+ve	Live	Sex	Status	Age	Comment
OX119	CTR1	19	X	X	M	CTR	36	Previously published (43)
NHDF1	CTR2	1	X	X	F	CTR	44	Previously published (44)
AH016	CTR3	3	X		M	CTR	80	
MK144	PD1	7	X		F	G2019S	57	Sister of JR036
MK002	PD2	4	X		F	G2019S	72	
JR036	PD3	1	X		M	G2019S	50	Brother of MK144

TH + ve: TH purified neurons.

Live: non-purified.

CTR: healthy control donor.

G2018S: PD patients carrying *LRRK2 G2019S* mutations.

M: Male.

F: Female.

**Figure 1.**
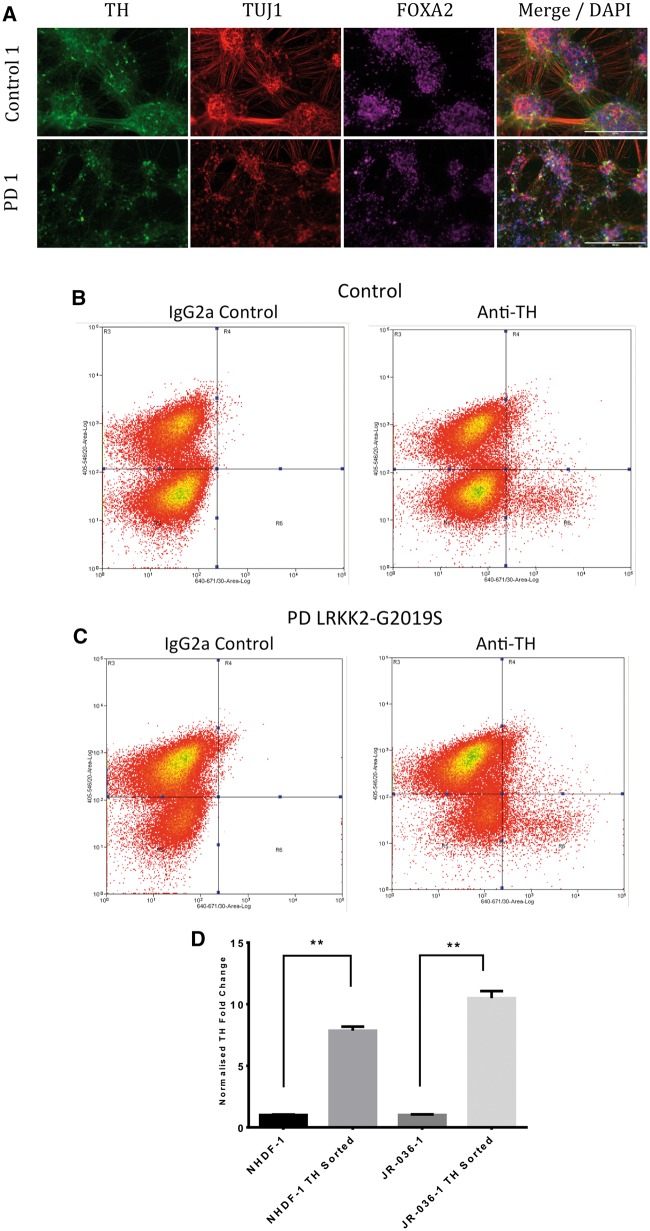
Purification of iPSC dopaminergic neurons by flow cytometry. (**A**) Representative immunostains of neurons demonstrates successful differentiation of control and PD samples in DaNs. (**B**) Representative FACS plots of the DA neuron isolation for control. (**C**) PD LRKK2-G2019S samples. Vertical axis denotes live/dead stain and horizontal axis TH- and TH+ cells. IgG2a was used as an isotype control. (**D**) Successful purification of TH positive neurons: qRT-PCR for TH expression on RNA extracts from sorted and unsorted control and LRKK2-G2019S lines.

In order to use RNA sequencing (RNA-seq) transcriptomics to investigate the potential mechanisms responsible for DaNs cell death in PD, we first purified the subpopulation of DaNs from within the heterogeneous differentiated cell population. Isolated DaNs were purified by fluorescence-activated cell sorting (FACS) using a live/dead stain, followed by fixation and staining with an antibody for TH to identify live, dopamine TH+ neurons (Fig. 1). To enable the use of the intracellular marker TH, cells were fixed and permeabilised to allow antibody entry. This also eliminated the induction of stress response genes following FACS, which might alter the transcriptomic profile (21). The IgG2a isotype control confirmed successful gating, and a clear population of live TH+ cells was observed in both control and *LRKK2-G2019S* cells (Fig. 1B and C). The number of cells collected from control and *LRKK2-G2019S* lines did not significantly differ from one another (Supplementary Material, Fig. S2A and B) and extracted RNA was of uniform high quality (RNA integrity analysis (RIN) >8) (Supplementary Material, Fig. S2C). Q-RT-PCR analysis of RNA extracted from unsorted and purified control and *LRKK2-G2019S* dopamine neurons confirmed the enrichment, with both control and *LRKK2-G2019S* sorted dopamine neurons displaying a 10-fold enrichment for TH compared to unsorted populations (Fig. 1D).

### Global expression profiles: purified iPSC-derived DaNs are highly similar to native mature LCM-isolated DaNs

Transcriptional profiles were generated from purified iPSC-derived DaN lines derived from six individuals: three PD patients carrying *LRRK2-G2019S* mutation and three controls. In order to evaluate the effect of the TH+ purification step on the transcriptional profiles, we generated additional RNA-seq data from two of the control lines prior to purification (Table 1) (Materials and Methods).

**Figure 2.**
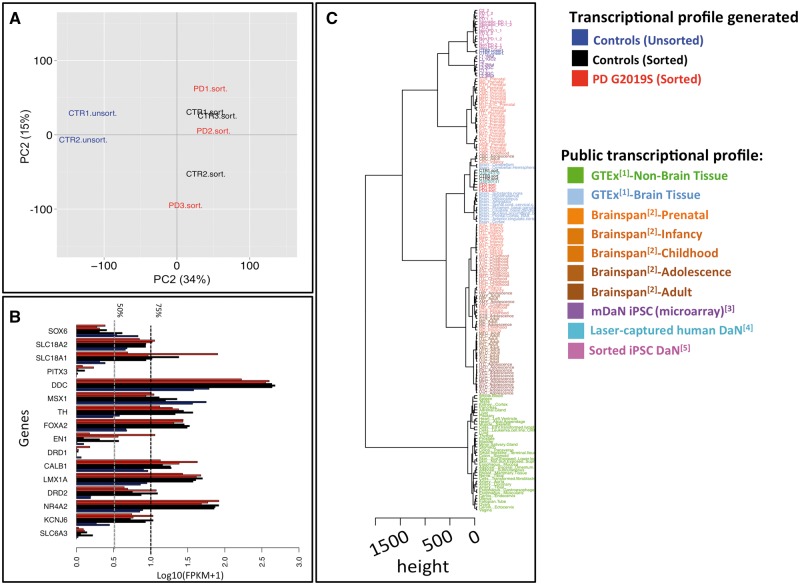
Transcriptomic evaluation of an iPSC-derived and purified model of dopaminergic neurons. (**A**) Principal component analyses performed from FPKM values of 17170 of 20157 protein coding genes for which the variance was different zero. x and y axis represent the principal component 1 and 2 explaining 34% and 15% of variance, respectively. (**B**) Expression level of 16 gene dopaminergic markers. The two vertical dotted lines represent the 50nd (gray) and 75nd (black) percentiles of expression level measure. (**C**) Comparisons of the transcriptional profiles of all eight iPSC-derived DaNs cell lines with the following publically-available transcriptional profiles: (i) RNA seq profiling generated from 53 human postmortem tissue profiles made available by the Genotype-Tissue Expression (GTEx) project (http://www.gtexportal.org/) (ii) RNA sequencing data profiling of up to sixteen cortical and subcortical structures across the full course of human brain development (http://www.brainspan.org/) (iii) microarray profiles of eight iPSC-derived unpurified DaNs cell lines including two controls lines (C1.1,C2), three lines carrying *LRRK2-G2019S* mutations (L1.1Mut, L2.3Mut, L2.2Mut) and three matching isogenic lines with engineered-corrections for *LRRK2-G2019S* mutation, isogenic line of L1.1Mut, L2.3Mut, L2.2Mut (L1.1GC2, L2.3GC, L2.2GC) (GSE43364, (23)) (iv) microarray profiles of two laser-captured human dopaminergic neuron dataset (GSE20141 & GSE24378). (v) RNA sequencing data profiling of 14 samples coming from of 7 iPSC derived DaN (two replicates by cell line) and FACs sorted by using a combination of surface markers (*CD133*, a stem/progenitor marker; *CD56*, a nerve cell adhesion molecule; *CD15* and *CD184*, *NSC* markers; and *CD24*, a cell differentiation antigen) derived from following subjects: (1) man with a five-year history of PD (PD) and heterozygous for *GBA-N370S* variant, (2) his monozygotic twin brother without PD (Non-PD), (3) one sporadic PD patient (Sporadic-PD) and (4) four control subjects (C) (GSE62642) (25) (Materials and Methods). The brainspan dataset uses the following acronyms: **URL** upper (rostral) rhombic lip; **VFC** ventrolateral prefrontal cortex; **DFC** dorsolateral prefrontal cortex; **LGE** lateral ganglionic eminence; **ITC** inferolateral temporal cortex (area TEv); **STC** posterior (caudal) superior temporal cortex (area TAc); **AMY** amygdaloid complex; **MFC** anterior (rostral) cingulate (medial prefrontal) cortex; **HIP** hippocampus (hippocampal formation); **CGE** caudal ganglionic eminence; **Ocx** occipital neocortex; **DTH** dorsal thalamus; **M1C-S1C** primary motor-sensory cortex (samples); **MGE** medial ganglionic eminence; **OFC** orbital frontal cortex; **PCx** parietal neocortex; **TCx** temporal neocortex; **M1C** primary motor cortex (area M1); **STR** striatum; **IPC** posteroventral (inferior) parietal cortex; **A1C** primary auditory cortex (core); **V1C** primary visual cortex (striate cortex); **S1C** primary somatosensory cortex (area S1); **CB** cerebellum; **MD** mediodorsal nucleus of thalamus; **CBC** cerebellar cortex

To assess the transcriptomic variation between the purified and non-purified lines, we performed principal component (PC) analyses using the FPKM values of 17,170 of the 20,157 protein coding genes for which the variance was not zero (Supplementary Material, Fig. S3). Across first principal component, which accounts for 34% of the total variance, we observed a clear separation between the transcriptomic profiles of the purified and the unpurified samples (Fig. 2A). This demonstrates that the cell-type purification step has increased transcriptomic uniformity in the purified samples. We did not observe that other factors such as patient age, gender or relatedness had a major impact on the transcriptional profiles (Supplementary Material, Figs S4 and S5).

Next, we examined the expression of marker gene transcripts, specifically eight major pluripotency marker genes (Supplementary Material, Fig. S6A), sixteen reference DaN marker genes (Fig. 2B) and three genes associated with PD (Supplementary Material, Fig. S6B). We found that all pluripotency markers exhibited low or undetectable expression levels with the exception of *SOX2*, which plays a role in the adult neurogenesis (22) and is known to be highly expressed in adult brain tissue (http://www.proteinatlas.org/ENSG00000181449-SOX2/tissue) (Supplementary Material, Fig. S6A). Twelve of sixteen dopaminergic markers were more highly expressed (highest quartile) in the purified iPSC-derived DaN lines as compared to the unpurified lines, reflecting successful purification of the DaNs (Fig. 2B). *GBA* and *SNCA*, genes involved in PD, were expressed in DaNs (Supplementary Material, Fig. S6B). As has been observed for other brain tissues, expression levels of the *LRRK2* gene were low but detectable (Supplementary Material, Fig. S6B). http://www.proteinatlas.org/ENSG00000188906-LRRK2/tissue).

Finally, we compared the transcriptional profiles of all eight sets of iPSC-derived DaNs with the following publically-available transcriptional profiles: (i) RNA-seq profiling generated from 53 human postmortem tissue profiles made available by the Genotype-Tissue Expression (GTEx) project (http://www.gtexportal.org/) (ii) RNA sequencing data profiling up to sixteen cortical and subcortical structures across the full course of human brain development (http://www.brainspan.org/) (iii) microarray profiles of eight iPSC-derived unpurified DaNs cell lines including two controls lines, three lines carrying the *LRRK2-G2019S* mutation and three matching isogenic lines with engineered-corrections for the *LRRK2-G2019S* mutation (GSE43364) (23) (iv) microarray profiles of two laser-captured human dopaminergic neurons dataset (GSE20141 & GSE24378) (24) (v) RNA sequencing data profiling seven iPSC-derived DaN lines (two replicates per line) and subsequently FACS sorted on a combination of surface markers (*CD133*, a stem/progenitor marker; *CD56*, a nerve cell adhesion molecule; *CD15* and *CD184*, *NSC* markers; and *CD24*, a cell differentiation antigen) derived from following subjects: (1) man with a five-year history of PD (PD) and heterozygous for *GBA-N370S* variant, (2) his monozygotic twin brother without PD (Non-PD), (3) one sporadic PD patient (Sporadic-PD) and (4) four control subjects (C) (GSE62642) (25).

Clustering all samples/tissues using the Euclidean distance matrix computed from the ranked transcript levels of 7305 common expressed protein-coding genes (Materials and Methods), showed that the six TH+-sorted iPSC-derived DaN lines exhibited transcriptional profiles highly similar to those obtained from mature laser-captured human dopaminergic neurons isolated from post-mortem tissue (Fig. 2C). However, the transcriptional profiles of the unsorted iPSC-derived DaN lines and those enriched for non-DaN-specific markers were found to cluster with prenatal brain (BrainSpan) tissues and with the previously-published and non-sorted iPSC-derived DaNs, indicating that cell-type heterogeneity is an important bias in unsorted iPSC-derived DaNs populations.

Taken together, the distinct expression profiles of the purified neurons compared to the unpurified cells, the increased expression of dopaminergic marker genes within the purified neurons, and the transcriptomic clustering of the purified neurons with adult nigral brain tissue, all support the conclusion that the TH+-purified iPSC-derived dopaminergic neuronal transcriptomic profiles are representative of mature native dopaminergic midbrain neurons. Thus, the TH+-purified cell populations enable studies focused to this particularly PD-relevant cell type.

### Genes differentially expressed between PD LRRK2-G2019S case and control iPSC-derived DaNs converge functionally

We performed differential expression analyses between the TH+-purified neurons (herein referred to as “purified”) from the three control lines and the three lines carrying the *LRRK2-G2019S* mutation. For this, we compared the variation in read counts per gene with DESeq2, a method demonstrably robust to logarithmic fold changes (LFC) of genes with low counts and appropriate for experiments with few replicates (26). Adjusting for gender and age, we found 40 differentially expressed (DE) genes with a False Discovery Rate (FDR) of 0.1 (Supplementary Material, Data S1). However, as we wished to look for functional convergence within shared molecular pathways amongst genes, rather than focus on each gene individually, we considered more DE genes by relaxing the *P*-value threshold at a cost of increasing the frequency of individual false positive genes. We considered those 168 DE genes with nominal *P*-value < 1% (Supplementary Material, Data S1) and tested for functionally-linked clustering within a phenotypic-linkage network (PLN) (27). Briefly, a PLN is constructed by evaluating and integrating multiple sources of gene functional information to create a gene network within which the distances between any pair of genes is inversely proportional to the likelihood that those genes influence the same mammalian phenotype (27). We found that 94 of 168 DE genes formed a significant functional cluster within the PLN (*P* < 10^−^^6^) (Supplementary Material, Fig. S7 and Data S1). While this functional clustering is generated by an amalgamate of functional genomics evidence, the 4 most prominent contributions made by Gene Ontology evidence include oxidative stress, glycosaminoglycans, immune response signalling (regulation of I-kappaB kinase/NF-kappaB signaling) and lipopolysaccharides.

We were concerned that the observed functional convergence within the PLN could simply be a result of selecting genes at random from the same cell type, and thus not a consequence of the *LRRK2-G2019S* mutation. Given the number of replicates, it was not possible to test this by permuting case/control status. Instead, we developed an alternative approach wherein we modelled each gene’s observed expression profile across the 6 lines and then randomly generated simulated transcriptomic profiles representing 10,000 sets of 3 case lines and 3 control lines (Materials and Methods). By examining the functional clustering amongst the most significantly differentially-expressed 168 genes in each simulated case/control comparison, we found that none of these simulations clustered as strongly as the 168 original DE genes (*P* < 10^−^^5^; Supplementary Material, Fig. S8) demonstrating that our signal was likely driven by *LRRK2-G2019S* mutation.

Among 168 DE genes observed, 109/168 genes had an increased expression level in the *LRRK2-G2019S* lines as compared to controls, while 59/168 genes had decreased expression levels. To validate the 168 DE gene set identified by RNA-seq (Supplementary Material, Data S1), six of the top most differentially expressed up- and down-regulated genes were selected for further qRT-PCR analysis. All the up-regulated (*HIST1H1A, ZNF441, SGCN, PTPRN2*) and the down-regulated (*RGCC, SV2B*) genes tested displayed relative expression similar to that identified in the RNA-seq of the purified PD *LRKK2-G2019S* patient DaNs compared to controls (Supplementary Material, Fig. S9).

**Figure 3.**
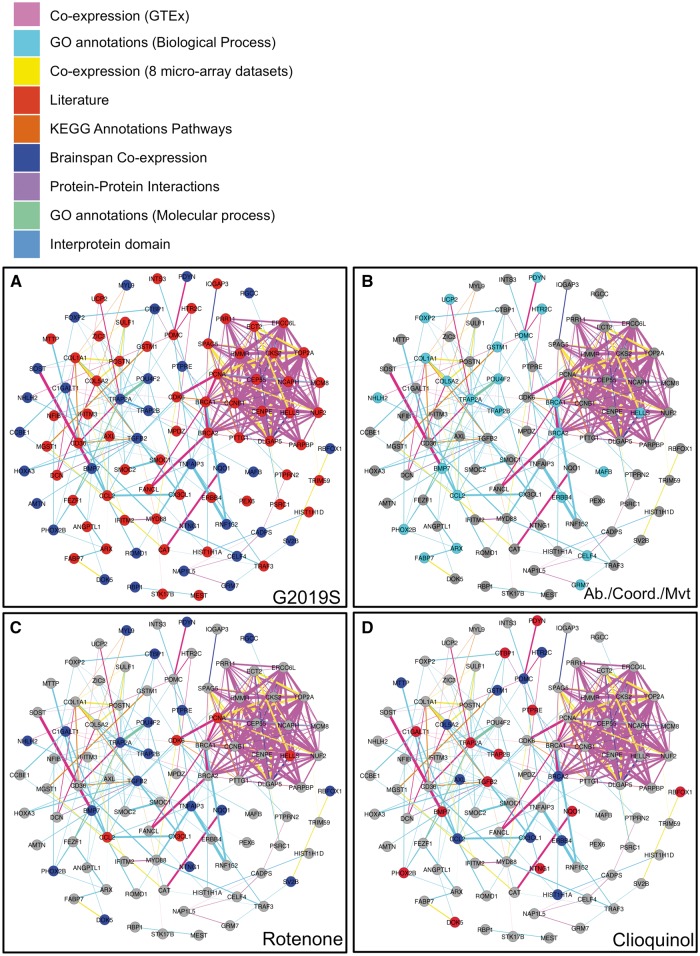
Functional associations within general PLN of 94/168 DE genes. The colour of links between genes indicates the most informative dataset for the relationship between gene pairs (see legend). The Panel (**A**) shows the relation between the up (red) and down (blue) regulated genes (reference control). The Panel (**B**) lists differentially expressed genes whose orthologue’s disruption in the mouse yields the phenotype abnormal capabilities/coordination/movement ((MP:0002066). The Panels C and D show genes for which expression is increased (red) or decreased (blue) after rotenone and clioquinol respectively by using top 1000 of up and dow regulated genes of CMAP rank matrix of each instance (Materials and Methods).

Furthermore by examining the network architecture of the 94 PLN-clustered DE genes, we noted that up- and, separately, down-regulated genes represented two distinct and significant clusters (Fig. 3A). Indeed, after separating the 168 DE genes by their direction of change, the set of 109 up-regulated genes and the set of 59 down-regulated genes each clustered significantly within PLN (respectively *P* < 10^−^^6^ (Supplementary Material, Fig. S7B) and *P =* 2.4×10^−^^4^ (Supplementary Material, Fig. S7C)). Furthermore, after randomly permuting the members of the up- and down-regulated gene sets we found no significant clustering within the randomly drawn sets (Supplementary Material, Fig. S7F) suggesting that these two groups of genes may represent distinct functional clusters and may be associated with distinct cellular/molecular perturbations induced by *LRRK2-G2019S* mutation.

To evaluate whether the observed 168 DE genes were associated with known molecular mechanisms underlying PD, we (i) looked for an overlap with known PD genetic risk factors, (ii) examined PD-relevant phenotypes associated with these genes’ mouse orthologues and (iii) examined publically-available drug transcriptional response profiles.

### Down-regulated genes are enriched in genes whose orthologous disruption in the mouse yields abnormal capabilities/coordination/movement phenotypes

We then examined whether the 168 DE genes were enriched for orthologues of mouse genes whose disruption yields relevant PD-relevant phenotype abnormalities; note that this information is not included within the PLN applied above (Materials and Methods). Considering those phenotypes that were within the 2 main categories of mouse phenotypes most relevant to PD, namely nervous system (MP:0003631) and behavior/neurological phenotypes (MP:0005386), we found significant enrichments of genes associated with abnormal nervous physiology phenotypes (MP:0003633) (q-value = 0.013,18.5 expected versus 29 observed genes) and abnormal motor capabilities/coordination/movement phenotypes (MP:0002066) (q-value = 0.037, 17.45 expected genes versus 28 observed genes) (Supplementary Material, Fig. S10 and Fig. 3B). For both observations, we found that the enrichment was largely driven by genes with a reduced expression in the *LRRK-G2019S* case lines, matching the direction of a dosage change associated with their mouse orthologues’ knock-out phenotypes (Supplementary Material, Fig. S11).

### Matching drug cellular response profiles to LRRK2-G2019S iPSC dopaminergic cellular profiles

A key aim following the identification of the molecular perturbations associated with disease is to identify therapeutics that might act to ameliorate those perturbations, thereby offering pathways to therapy. For this, we used the Connectivity Map (CMAP) resource to identify drugs that influence the expression of the 168 DE genes (https://www.broadinstitute.org/cmap) (28,29). CMAP holds the cellular transcriptomic response profiles recorded following the exposure of cells to over 1,000 compounds and allows these profiles to be matched against user-provided transcriptomic profiles to identify compounds that provoke a correlated or anti-correlated transcriptomic response. Given a disease-associated transcriptomic profile, compounds that provoke a correlated profile may provide insights into disease-relevant processes or new disease models, while compounds that provoke an anti-correlated response may themselves be of therapeutic value or provide lead to identifying new therapies (29,30).

Examining the significant results reported by interrogating CMAP with our 168 DE gene disease signature, we observed that rotenone, a compound known to induce PD (31), induces a gene expression profile significantly similar to that observed within the *LRRK2-G2019S* cells lines (enrichment score =0.284, *P =* 7.6 ×10^−^^3^ and Supplementary Material, Data S2 and Fig. 3C). A compound generating a significantly counteracting gene expression profile was clioquinol, a drug known to rescue dopamine neuron loss and Parkinsonian behavioural phenotypes in mouse models (enrichment score = −0.557, *P =* 2.7 ×10^−^^3^; see Supplementary Material, Data S2 and Fig. 3D) (32–35)

To test the hypothesis that these drug signatures were not associated with genes generally expressed in dopaminergic neurons, but rather were specific to those genes we identified as differentially expressed between the PD *LRRK2-G2019S* case and control cell lines, we re-interrogated CMAP manually by hand with 20 sets of simulated 168 DE genes as used above to similarly test the clustering within the PLN (Materials and Methods) but found that no simulated set displayed matched significantly to these drug signatures reassuring that their relevance is derived from the PD *LRRK2-G2019S* cellular perturbation (Supplementary Material, Table S1).

### Unpurified iPSC-derived LRRK2 DaNs transcriptomic profiles do not yield functional, genetic or molecular associations

To compare the transcriptomic signature revealed by purifying DaN cells to that obtained from an heterogeneous iPSC midbrain neuronal population, we compared our results to those obtained from a previous gene microarray transcriptional study (GSE43364) (23) using three unpurified iPSC-derived dopaminergic midbrain neurons carrying *LRRK2-G2019S* mutations and their respective isogenic controls. The profiles of iPSC DaN lines from this study cluster with those from our unsorted lines supporting the relevance of the comparison (Fig. 2). Using the approach here to perform DE analyses, we found only one gene differentially expressed at an FDR < 0.10 in the unsorted lines (gene *ZKSCAN5*; Supplementary Material, Data S2). As with the transcriptomic analyses of the purified DaN lines above, we considered those DE genes with nominal *P*-value < 1% yielding a total of 85 DE genes; 30 up-regulated in *LRRK2-G2019S* lines and 55 down-regulated (Supplementary Material, Data S3). Only the gene *NHLH2* was found to be differentially-expressed at this threshold in both the Reinhardt *et al.* study and our study.

We repeated then the same network/genes enrichment/drug signatures analyses described above. Unlike the DE genes between purified iPSC derived DaN lines, we did not observe that this set of 85 DE genes significantly functionally clustered within the same PLN (*P =* 0.21, 0,08 0.16 for all, up-regulated and down-regulated genes respectively) nor were these 85 DE genes enriched for mouse orthologous whose disruption was associated with abnormal nervous physiology phenotypes (MP:0003633) or abnormal motor capabilities/coordination/movement phenotypes (MP:0002066) (*q-values* of 1 and 0.98 respectively). Lastly, by comparison to CMAP drug response profiles, we did not observe a significant intersection with either rotenone, clioquinol nor any other agent known to reverse or induce PD (Supplementary Material, Data S4).

As the analyses of global expression showed that unpurified iPSC derived DaNs are dominated by immature neuronal populations (Fig. 2), the cellular heterogeneity is likely cause of these negative results. Taken together, these results support a conflation of the gene expression profiles obscuring differential expression studies.

## Discussion

PD is a complex disorder, and we have little knowledge of the exact mechanisms behind the associated dopaminergic neuron susceptibility and death. In this study, we present a method for purifying iPSC-derived dopaminergic neurons, which allows methods such as RNA-sequencing to study this PD-relevant cell type specifically. Once purified, we show that the transcriptomic profiles of iPSC-derived dopaminergic neurons appear highly similar to those obtained from post-mortem mature dopaminergic neurons isolated through LCM. Purified dopaminergic neurons derived from PD patients carrying *LRRK2-G2019S* variants revealed a novel set of genes whose expression is perturbed as compared to models derived from controls. Most notably, the gene expression variation was found to be correlated with variation reportedly induced by rotenone, a compound that causes drug-induced Parkinsonism.

When comparing the phenotypic profiles of purified and unpurified dopaminergic neurons, the purified dopaminergic neuronal population obtained by FAC sorting on a DaN-specific marker displayed the transcriptomic profile of a mature midbrain dopaminergic neurons (Fig. 2). By contrast, the transcriptomic profiles of unpurified heterogeneous midbrain neuronal populations from both this study and a previous study (36), and those from a study selecting cells on non-DaN-specific markers (25), cluster with immature prenatal neurons, suggesting a dominating contribution of immature neurons which limits the insights into the PD-relevant dopaminergic neuronal population and emphasizes the importance of selecting the DaNs specifically from the populations prior to RNA-seq analysis. While the use of an intracellular marker (TH) for cell sorting kills the cells, exposing the DaN gene expression profiles offers significant insights, as we demonstrate here. Furthermore, although two purified dopaminergic neuronal lines (CTR1-sort. and CTR 2-sort.) are derived from the same control individuals as the two unpurified lines (CTR1-unsort. and CTR2-unsort.), their transcriptional profiles were more similar to the other purified lines from different individuals, suggesting that cellular heterogeneity has a greater impact on the bulk transcriptional profile than the genetic background. Thus, isolating cell types may be more important than isogenic controls for particular study designs.

In a novel approach to investigate how particular drugs may influence the expression of genes affected by the PD *LRKK2-G2019S* mutation, CMAP identified the mitochondrial complex I inhibitor, rotenone, as the compound with an expression profile most analogous to the 168 DE genes. Rotenone is one of the most extensively utilized chemical models of PD, with chronic exposure to rotenone causing a highly selective nigrostriatal dopaminergic degeneration that is associated with motor impairment in drosophila, mouse and rat models of PD (31,37–39). The identification of a compound so comprehensively linked to PD as that most related to the 168 DE gene expression profile further supports the iPSC-derived FACS sorted DaNs as a highly relevant model for PD.

The finding of a strong hit for clioquinol as a compound that induces a transcriptomic response anti-correlated to that observed within the PD *LRRK2-G2019S* dopaminergic neurons is intriguing and a robust validation of our methodology. Clioquinol is an iron/copper/zinc chelator and anti-oxidant previously used extensively as an antibiotic and antimalarial. It was shown in 2003 to prevent dopaminergic cell death in the MPTP toxin mouse model of Parkinson’s, most likely through a reduction in reactive iron (35). More recently, clioquinol has been shown to rescue cognitive and motor function, and dopamine neuron loss in α-synuclein hA53T transgenic mice (34) and in microtubule associated protein tau knockout (*Mapt-/-*) mice (32). Clioquinol has also shown promise in the treatment of mouse models of Alzheimer’s disease (AD) (40) and has been used in a small Phase 2 trial for AD in which it was well tolerated and appeared to reduce cognitive decline (41). Other anti-malarials (amodiaquine and chloroquine) have been also found to alleviate behavioural deficits within a 6-hydroxydopamine lesioned rat model of PD (42). Taken together, the data build a strong case for clioquinol as a therapeutic molecule for Parkinson’s and validate our approach for identifying candidate drugs for repurposing.

Dopaminergic neurons do not exist in the brain in isolation, they form defined functional neuronal circuits existing in the context of a complex mix of supporting glial cells. Our work here has focussed on dopaminergic neuronal cultures as a tractable model to understand transcriptional perturbations in the most vulnerable cell type in Parkinson’s disease. Interestingly, *LRRK2* is highly expressed in astrocytes and further studies will be needed to investigate transcriptomic changes in mixed dopamine neuron/astrocyte co-culture models of disease.

In summary, this study demonstrates the ability of iPS cells, when combined with appropriate experimental controls, to deliver in vitro cellular disease models whose gene expression profiles are extremely similar to inaccessible mature neuronal cell types. Despite comparing only three lines derived from PD patients carrying *LRRK2-G2019S* variants to three controls lines, the differential gene expression pattern identified is both correlated with the effects of PD-inducing compounds and anti-correlated to the effects of compounds found to have efficacy in alleviating PD symptoms in rodent models. The approach described here identifies molecular perturbations to direct future cellular phenotyping studies to understand disease mechanisms, and allows the identification of potential re-purposable drugs for disease treatment.

## Materials and methods

### Participation recruitment

Participants were recruited to this study having given signed informed consent, which included mutation screening and derivation of hiPSC lines from skin biopsies (Ethics committee: National Health Service, Health Research Authority, NRES Committee South Central – Berkshire, UK, who specifically approved this part of the study - REC 10/H0505/71).

### LRKK2-G2010S mutation screening

Parkinson's disease patients and controls from the Discovery clinical cohort established by the Oxford Parkinson’s Disease Centre (OPDC) were screened for the presence of *LRRK2-G2019S* heterozygous mutation and excluded for other known PD-related mutations (Supplementary Material, Fig. S1A). Genomic DNA was extracted from iPSC-derived cells using Illustra tissue and cells genomic miniprep kit and quantified using a Nanodrop 1000. Polymerase chain reaction (PCR) was carried out using AmpliTaq DNA polymerase with primer sequences 5′-TTTAAGGGACAAAGTGAGCAC-3′ and 5′-ACTCTGT�TTTCCT�TT�TG�ACTC-3′. The PCR product was digested using the restriction enzyme SfcI and the product analysed for the presence of the G2019S mutation by agarose gel electrophoresis.

### Generation of human iPSCs

Induced pluripotent stem cell (iPSC) lines were derived from three PD patients carrying a *LRRK2-G2019S* heterozygous mutation, and three healthy control individuals, from the Oxford Parkinson’s Disease Centre (OPDC) Discovery Cohort (Table 1).

Skin punch biopsies were obtained from participants and low passage fibroblast cultures established and transduced with reprogramming retroviruses (c-MYC, KLF4, SOX2, OCT3/4 and Nanog). Colonies displaying iPSC morphology were picked and passaged on MEFs by manual dissection before conversion to feeder-free culture. One control line and the three patient lines are described here for the first time, and full characterization information is given, with full details of characterization methods given in Supplementary Material, Notes S1and S2. The other two control iPSC lines, iPS-OX1-19 and iPS-NHDF-1 have been described fully elsewhere (43,44).

### Characterisation of iPSC

hiPSC adapted to feeder-free culture on Matrigel (BD Biosciences) were banked in bulk and harvested for characterisation analyses. RNA and genomic DNA were made using an All-Prep kit (Qiagen). Genome integrity and cell line identity were confirmed by a high resolution Illumina CytoSNP-array, silencing of retroviral transgenes upon establishment of pluripotency was confirmed by quantitative RT-PCR, and their gene expression profiles conformed to those of benchmark human pluripotent stem cell lines as assessed by Pluritest. All iPSC lines displayed embryonic stem cell-like morphology and expressed the pluripotency-associated protein TRA-1-60 (Supplementary Material, Fig. S1D).

Further details on fluorescence activated cell sorting (FACs), qRT-PCR for assessing transgene silencing, Illumina Human CytoSNP-12v2.1 beadchip array for assessing genome integrity and tracking, and Illumina HT12v4 transcriptome array for assessing conformity to benchmark pluripotent expression profiles (PluriTest), can be found in Supplementary Material, Note S2.

### Generation of human iPSC-derived dopamine neurons

In order to study gene expression profiling in those cells most vulnerable to death in *LRRK2-G2019S* models of Parkinson’s disease, iPSCs from patients harbouring these mutations and controls needed to be differentiated into dopaminergic (DA) neurons. Three control (NHDF-1, OX1-19 and AH-016-3) and three *LRRK2-G2019S* (JR-03601, MK002-4 and MK144-7) patient lines were differentiated, as described previously by Kriks *et al.* (20), with slight modifications. Cells first undergo 21 days of patterning and differentiation, are replated and are then matured for a further 2 weeks, resulting in cells at days in vitro (DIV 35) when collected for flow cytometry.

### Characterization of iPSC-derived DaNs cultures

Control and PD *LRKK2-G2019S* lines successfully differentiated into dopaminergic neurons, as assessed by positive *TH* (dopaminergic marker), *FOXA2* (dopaminergic marker) and b-3 tubulin/TUJ1 (neuronal marker) staining by immunofluorescence (Fig. 1A). Additionally, post-differentiation, the PD *LRKK2 G2019S* lines display an increase in midbrain dopaminergic markers; *LMX1A*, *FOXA2*, *TH*, *PITX3*, *GIRK2* and *NURR1* by qPCR compared to iPSCs. Conversely, they also display a decrease in *OCT4*, a marker of stem cell pluripotency (Supplementary Material, Fig. S1F).

### Quantitative real-time PCR and immunocytochemistry

RNA was extracted from cells using the RNeasy Micro kit (QIAGEN) as per manufacturer’s instructions and quantified using a NanoDrop 1000 (ThermoScientific). cDNA was synthesised using Superscript III reverse transcriptase (Life Technologies) as per manufacturer’s instructions. Quantitative real-time PCR was carried out using Fast SYBR Green Mastermix on a StepOnePlus thermal cycler (Life Technologies).

For immunostaining, cells on coverslips were fixed in 4% paraformaldehyde then stained in PBS/0.1% TritonX as follows. Cells were blocked with 10% donkey serum for 1 h then incubated with the following primary antibodies: TH (1:500, Millipore), TUJ1 (1:500, Covance), FOXA2 (1:250, R&D Systems) in 1% donkey serum for 1.5 h, then secondary antibodies in 1% donkey serum for 1 hr. Finally, cells were incubated with 1 µg/mL DAPI for 10 min at room temperature, cells were then washed 1x PBS before mounting onto slides and imaged using an EVOS FL-Auto microscope.

### Purification of iPSC derived live/DaNs by flow cytometry

Control and PD *LRKK2-G2019S* lines, seeded at 400,000/cm2 were washed with 2ml of warm DPBS and dissociated with 300 µl of warm trypsin-EDTA for 5 min. During incubation a 40 um cell strainer was primed with 1ml cold PBS + 3U/ml DNAse I. The trypsin was inhibited with 400 µl of warm defined trypsin inhibitor + 6U/ml DNAse I and cells passed through the cell strainers into BSA pre-coated 50 ml falcon tubes. The cell strainers were washed with 1ml cold PBS + 3U/ml DNAse I and spun for 300 *g* for 5 min at 4 °C. The supernatant was aspirated and pellets kept on ice for the remainder of the protocol,

Pellets were re-suspended in 1ml cold PBS, transferred to a protein lo-bind tube and spun for 300 *g* for 3 min at 4 °C, The supernatant was aspirated and the cells re-suspended in 1 ml Live/dead yellow fixable stain (1:500) in PBS, kept on ice for 10 min in the dark, followed by the addition of 200 µl of 10% BSA in PBS and cells spun for 300 *g* for 3 min at 4 °C, Cell pellets were then re-suspended in 30 µl PBS and 200 µl of 4% paraformaldehyde (PFA) in PBS added to the cell suspension and incubated on ice for 10 min, after which 15 µl of 2M Glycine in PBS was added to inhibit PFA fixation. To prime the cells for permeabilisaiton, 100 µl of permeabilisation buffer ((PB) (0.1% saponin, 2% BSA, 2M DTT, 100U/ml RNAse inhibitor, 2ug/ml normal goat IgG, PBS)) was added and cells spun at 300 *g* for 3 min at 4 °C,

Cells were then permeabilised with the addition of 200 µl of PB, kept on ice for 10 min and the samples then split into two tubes; Tube A (33 µl) and Tube B (166 µl). Both tubes were then incubated with PB containing the appropriate primary antibody; Tube A (100 µl normal mouse IgG2a antibody (1:500)) and Tube B (500 µl Mouse anti-TH F-11 antibody (1:2000)), rotating in the dark at 4 °C for 1 h. Cells were spun at 300 *g* for 3 min at 4 °C and then re-suspended in PRDB buffer (2% BSA, 5mM DTT, 100 U/ml RNAse inhibitor, PBS) containing secondary antibody (Goat anti-mouse Alexa 635 (1:2000)) and kept on ice for 15 min in the dark

Cells were then spun at 300 *g* for 3 min at 4 °C, washed with 200 µl PRDB buffer and then resuspended in 400 µl PRDB buffer before being transferred to polypropylene tubes and Flow cytometry sorted. Samples were sorted into 2ml Eppendorf DNA LoBind tubes containing 100 µl PRDB buffer + 2 µl RNAse inhibitor. Post FACS, samples were spun at 1000 *g* for 5 min at 4 °C, re-suspended in 300 µl RLT buffer (Qiagen RNeasy micro extraction kit) + 1% beta-mercaptoethanol (BME) and vortexed for 5 min, followed by incubation for 20 min at room temperature. Samples were then stored at −80 °C until RNA extraction.

### RNA extraction

RNA was extracted from the sample pellets using an RNeasy micro kit (Qiagen) with minor alterations. Briefly, 580 µl of RNase-free water and 20 µl of 10 mg/ml Proteinase K was added to the cells and incubated at 55 °C for 10 min. After the addition of 450 µl of 100% ethanol, the cell suspension was loaded onto MinElute spin columns in a 2 ml DNA LoBind collection tube and spun at 8000 *g*for 15 s. Pellets were washed with 350 µl Buffer RW, centrifuged at 9000 *g*for 15 s and flow-through discarded. 10 µl of DNAse I + 70 µl of RDD buffer were mixed before being added to the column, incubated for 20 min at room temperature, after which 350 µl of RW1 buffer was added and cells spun at 9000 *g*for 15 s.

Cells were washed with 500 µl Buffer RPE, centrifuged at 9000 *g*for 15 s, then further washed with 500 µl 80% ethanol before being centrifuged at 9000 *g*for 5 min to dry the silica-gel membrane. The column was transferred to a new 1.5 ml DNA LoBind collection tube, 12 µl of RNAse-free water was added, incubated for 10 min and the column centrifuged at maximum speed for 1 min to elute the RNA. 1.2 µl of RNA was removed for concentration and RNA integrity analysis (RIN analysis) on a bioanalyzer

### RIN analysis and RNA quantitation

RNA integrity (RIN) and concentration was analysed on a 2100 bioanalyzer system (Agilent), utilizing the RNA 6000 pico kit (Agilent), as per manufacturer’s instructions. The samples were diluted before being loaded onto the bioanalyzer; JR-03601 (1:5), NHDF-1 (1:4), MK002-4 (1:4), OX1-19 (1:10), MK144-7 (1:4) and AH-016-3 (1:5).

### RNA library construction and sequencing

The polyadenylated fraction of RNA isolated from eight samples was used for 100bp paired-end RNA-seq with coverage 43.8 ± 4.1 sd million pairs read per sample. We used the SMARTer^®^ Ultra™ Low RNA Kit for Illumina^®^ Sequencing (Clontech) followed by the NEBNext^®^ DNA Library Prep Master Mix Set for Illumina^®^ to contruct poly(A) selected pair-end sequencing libraries. Both kits were used as per the manufacturer’s instructions except that published in-house custom indexes were used (45). The resulting multiplexed libraries were sequenced using Illumina TruSeq v3 chemistry. After indexing, all samples were combined into a single library and sequenced on two lanes of an Illumina HiSeq 2000 System.

### Quality control of RNA sequence data

By checking the quality of RNA sequencing data using the FASTQC software (version 0.9.3) (http://www.bioinformatics.babraham.ac.uk/projects/fastqc/) via the CGAT pipeline pipeline_readqc.py, we observed (i) an abnormal GC content and per base GC content for 5’ reads due to Hexamer priming bias (46) (Supplementary Material, Table S2) and (ii) an overrepresentation of some RNA sequence due to adapter contamination (Supplementary Material, Table S2 and Fig. S12).

### Exons genes annotations file

We generated annotations within the ENSEMBL gene set after reconciliation with the UCSC genome assembly from human genome (hg19) by using the CGAT pipeline pipeline_annotations.py. Annotations here are the original ENSEMBL annotations bar some filtering. The gtf file generated provided the information regarding exon parts of transcripts. This set includes both coding and non-coding transcripts. Coding transcripts span both the UTR and the CDS

### Alignment and quality control of alignment

We aligned our RNA sequences to the human genome (hg19) using STAR (version 2.3.0) (47). STAR is a mapper developed for RNA-seq data and is able to ignore adapters by clipping. We generated the index required by STAR using the following options:

–runMode genomeGenerate
–genomeFastaFiles genome softmasked fasta file (hg19)
–sjdbGTFfile gtf containing all known gene models (generated with CGAT pipeline pipeline_annotations.py)
–outFilterType BySJout

We aligned reads with the CGAT pipeline pipeline_mapping.py (option: make mapping) using STAR default options and:

- runMode alignReads
- genomeLoad LoaDaNsdRemove
- outStd SAM
- outSAMstrandField intronMotif
- outSAMunmapped Within
- outFilterType BySJout

The two bam files of each of the eight libraries coming from two lanes were then merged by using samtools (version 1.8) (48) by running the CGAT pipeline pipeline_mapping.py with the option make mergeBAMFiles. We compiled the statistic regarding the quality of mapping by using make buildBAMStats of CGAT pipeline pipeline_mapping.py. We successfully mapped uniquely 29.8 +/- 3.0 sd millions of pair reads (Supplementary Material, Table S3). However we observed a non-uniform read coverage biased to the 3’ end of genes (Supplementary Material, Fig. S13).

### Counts read overlapping exons annotations

The number of reads overlapping a gene were computed using HTSeq count (version 0.6.1p1) (49) with the intersection-strict mode. The sam files were sorted by read name with samtools. The exon annotations file was generated with the CGAT pipeline pipeline_annotations.py.

### Fragments per kilobase of exon per million fragments mapped (FPKM)

The FPKM values of eight libraries were computed by using cuffquant (versions 2.2.1) on bam files and exons annotations file to first compute gene and transcript expression in RNA-seq samples. We then used cuffnorm (version 2.2.1) to compute the FPKM values normalized across all eight RNA-seq libraries (50).

### List of protein coding genes

The list of 20,157 protein coding genes was obtained from the UCSC genome browser for the hg19 assembly by using the following query:

SELECT T1.name FROM ensGene T1, ensemblSource T2 WHERE T1.name = T2.name and source=“protein_coding”

### Principal component analyses

We performed principal component analyses by using the function prcomp in R (R version 3.1.2) with the following parameters: center = TRUE and scale = TRUE. We used the FPKM values of 20157 protein coding genes in eight samples. 2857 protein coding genes were discarded because variance equals to zero.

### Public transcriptional genes profiles

We compared the transcriptional profiles of iPSC DaNs with following public transcriptional profile:

1. RNA seq profiling generated from 53 human postmortem tissue profiles made available by the Genotype-Tissue Expression (GTEx) project (http://www.gtexportal.org/, ((51), http://www.gtexportal.org/, dbGaP Study Accession: phs000424.v5). We computed for each gene in each tissue the average of FPKM value.
2. RNA sequencing data profiling of up to sixteen cortical and subcortical structures across the full course of human brain development (http://www.brainspan.org/) (52). From the expression matrix in RPKM values, we computed the average of expression for stage: Prenatal (2pcw-38pcw), Infancy (Birth - 18 months, Childhood (19 months to 11 years), Adolescence (12–18 years), Adult (19–60 years+) and for the following cortical and subcortical structures: URL upper (rostral) rhombic lip VFC ventrolateral prefrontal cortex DFC dorsolateral prefrontal cortex LGE lateral ganglionic eminence ITC inferolateral temporal cortex (area TEv STC posterior (caudal) superior temporal cortex (area TAc) AMY amygdaloid complex MFC anterior (rostral) cingulate (medial prefrontal) cortex HIP hippocampus (hippocampal formation) CGE caudal ganglionic eminence Ocx occipital neocortex DTH dorsal thalamus M1C-S1C primary motor-sensory cortex (samples) MGE medial ganglionic eminence OFC orbital frontal cortex PCx parietal neocortex TCx temporal neocortex M1C primary motor cortex (area M1 STR striatum IPC posteroventral (inferior) parietal cortex A1C primary auditory cortex (core) V1C primary visual cortex (striate cortex S1C primary somatosensory cortex (area S1 CB cerebellum MD mediodorsal nucleus of thalamus CBC cerebellar cortex
3. microarray profiling of eight iPSC-derived DaNs cell lines including: two control lines (C1.1,C2), three lines carrying on mutation *LRRK2-G2019S* (L1.1Mut, L2.3Mut, L2.2Mut), three lines corrected for *LRRK2-G2019S* mutation, isogenic line of L1.1Mut, L2.3Mut, L2.2Mut (L1.1GC2, L2.3GC, L2.2GC) (GSE43364) (23). We used the normalized and background subtracted intensity value.
4. microarray profile of two laser-captured human dopaminergic neuron dataset (GSE20141 & GSE24378). For each dataset, we downloaded the raw intensity values by using the R library, GEOquery, performed a Robust Multi-array Average transformation on probe-level with function RMA of R library affy, and computed the expression by gene by using the mean of expression probe measures. We took the median normalized expression value across replicates as the single expression gene measure.
5. RNA sequencing data profiling of 14 samples coming from of 7 iPSC derived DaN (two replicates by cell line) and FACs sorted by using a combination of surface markers (*CD133*, a stem/progenitor marker; *CD56*, a nerve cell adhesion molecule; *CD15* and *CD184*, *NSC* markers; and *CD24*, a cell differentiation antigen) derived from following subjects: (1) man with a five-year history of PD (PD) and heterozygous for *GBA-N370S* variant, (2) his monozygotic twin brother without PD (Non-PD), (3) one sporadic PD patient (Sporadic-PD) and (4) four control subjects (C) (GSE62642) (25). We used the FPKM values as expression measures generated by this study (25).

### Method to compare the transcriptional profiles

We discarded protein-coding genes that were not expressed in one of set used to perform these comparisons: 7,305 protein coding genes were used in the comparisons for 162 tissues/cells. Expecting that the distributions of gene expression level were different between sets (array, RPKM, FPKM), we applied a non-parametric approach by ranking the genes according their expression level in each tissue/cell. From this rank matrix (row: tissue/cell, row: gene), we computed the Euclidean distance matrix between tissue/cell and performed clustering analyses by using Ward’s hierarchical approach.

We noticed that samples cluster according to their tissue specificity and not just according to tissue set. For example, the brain tissues described within GTeX cluster with BrainSpan tissues, rather than with the non-brain tissues within GTeX.

### Differential expression analyses

Differential expression analyses were performed with DESeq2 (version 1.6.3) (26) from the number of reads per protein coding gene using a multifactorial design that included the sex and age of individuals.

### Phenotypic linkage network

We updated the general PLN developed by Honti *et al.* (27) by integrating two new co-expression dataset based on RNA sequencing experiment coming from brainspan project (52) and GTEx project (51). Each genomic dataset was evaluated on its ability to predict the similarity of phenotypes observed following disruption of these genes’ unique orthologues in the mouse (Supplementary Material, Table S4). From this evaluation on a unique phenotypic benchmark, we scaled each individual dataset and combined their information into a single gene-pair measure. To remove spurious associations and increase computability, we considered only the 1,000,000 highest weighted gene links.

### Testing for functional clustering in PLN

To evaluate whether the DE gene set demonstrated unusually similar functionality, we examined the extent to which those genes clustered within the PLN. For this, we compared the sum of weighted links observed between these genes as compared to an equal number of random genes. To avoid detection of a gene cluster that reflected only a set of genes expressed in iPSC DaNs, we matched the randomized gene on CDS length, on degree in the PLN and on FPKM values to the set of DE genes. Specifically, within the PLN, we observed a median node degree of 117 (mean 175) for the 168 DE genes while our matched randomised gene set had a median of 118 (mean 176). For the clustering, we observed 330 links amongst the 168DE but only 177 links on average amongst random gene sets matched for degree and CDS length.

### Generation of random set of read counts

We simulated transcriptome profiles for 10,000 genes set for three cases and three controls. We generated read counts for 10 million genes from a negative binomial distribution where parameters *µ* and φ were sampled from the joint distribution of estimates from observed counts. We performed these simulations by using the R framework NBSim developed by Zhou *et al.* (53), http://imlspenticton.uzh.ch/robinson_lab/edgeR_robust) *et al.* with following options:

foldDiff = 3

nTags = 1000000

add.outlier = TRUE

outlierMech = “S”

pOutlier = 0.01

drop.extreme.dispersion = 0.1

The parameters were selected to get similar QQ plot profiles compared to QQ plot profiles of the nominal *P*-value of differential expression analyses performed with DESEq2 on protein coding genes read counts of 3 sorted control lines versus 3 sorted *LRRK2-G2019S* lines.

We then generated a random set of read counts for each gene by matching the original gene set on the mean of counts computed on six sorted lines used in our differential expression analyses.

### Enrichment test for differentially expressed genes whose orthologues’ disruption in the mouse yields an abnormal phenotype

To perform an enrichment test in orthologous mouse genes with phenotype annotation (e.g. x genes with y annotations), we considered all orthologous genes with a mouse phenotype and compared the number of DE genes with a specific mouse phenotype annotation to the number of random genes with same phenotype annotations, generated by considering the same number of de genes (among DE genes with mouse annotations) and by matching the original gene set for (1) the CDS length and gene expression (2) the expression level estimated in FPKM measure in control purified lines.

### Identification of genes for which their expression level is the most impacted in different CMAP instances by the same perturbagen

To identify the genes among DE genes for which their expression level were the most impacted in the same way in different CMAP instances by the same perturbagen, we used the matrix of rank of CMAP. In this matrix the CMAP instances are ranked according to their connectivity score for each probe set. We identified the top 1000 of up-regulated and down-regulated probe. After mapping probe set to gene, we considered that gene was up (or down)-regulated when all its probes in all instances were up (or down)-regulated.

### Differential expression analyses of a public microarray transcriptional dataset assayed on non-purified iPSC-derived DaNs cell lines

To evaluate the effect of the purification step to remove the noise caused by cellular heterogeneity on the differential expression gene results in the context of iPSCs derived DaNs cells populations, we compared our results with those of a previous gene microarray transcriptional study (GSE43364) (23) done on iPSC-derived unpurified DaNs cell lines. We performed differential expression analyses between three lines carrying on *LRRK2-G2019S* mutation (L1.1Mut, L2.3Mut, L2.2Mut) and three lines corrected for *LRRK2-G2019S* mutation, isogenic line of L1.1Mut, L2.3Mut, L2.2Mut (L1.1GC2, L2.3GC, L2.2GC) by using the limma R bioconductor package. The full R code used is given in the Supplementary Material, Note S3. The full differential expression results are represented in the Supplementary Material, Data S3. 85 genes was associated with nominal *P*-value < 1%.

## Accession number

The SNP datasets and the Illumina HT12v4 transcriptome array results have been deposited in the Gene Expression Omnibus (GEO) under accession number SuperSeries GSE77664: the SNP data series is GSE77662; the expression data series is GSE77663.

## Supplementary Material

Supplementary Material is available at *HMG* online.

## Supplementary Material

Supplementary Data
