# Supplementary material for: Transcriptomic profiling of purified patient-derived dopamine neurons identifies convergent perturbations and therapeutics for Parkinson’s disease
Source: Hum Mol Genet. 2017 Jan 17;26(3):552–66. doi: 10.1093/hmg/ddw412 (PMC5409122; doi:10.1093/hmg/ddw412)
Supplement: Supplementary Data [file ddw412_Supp.zip › ddw412-suppl_data/Supporting_Information_LRRK2_HMG.docx]

**
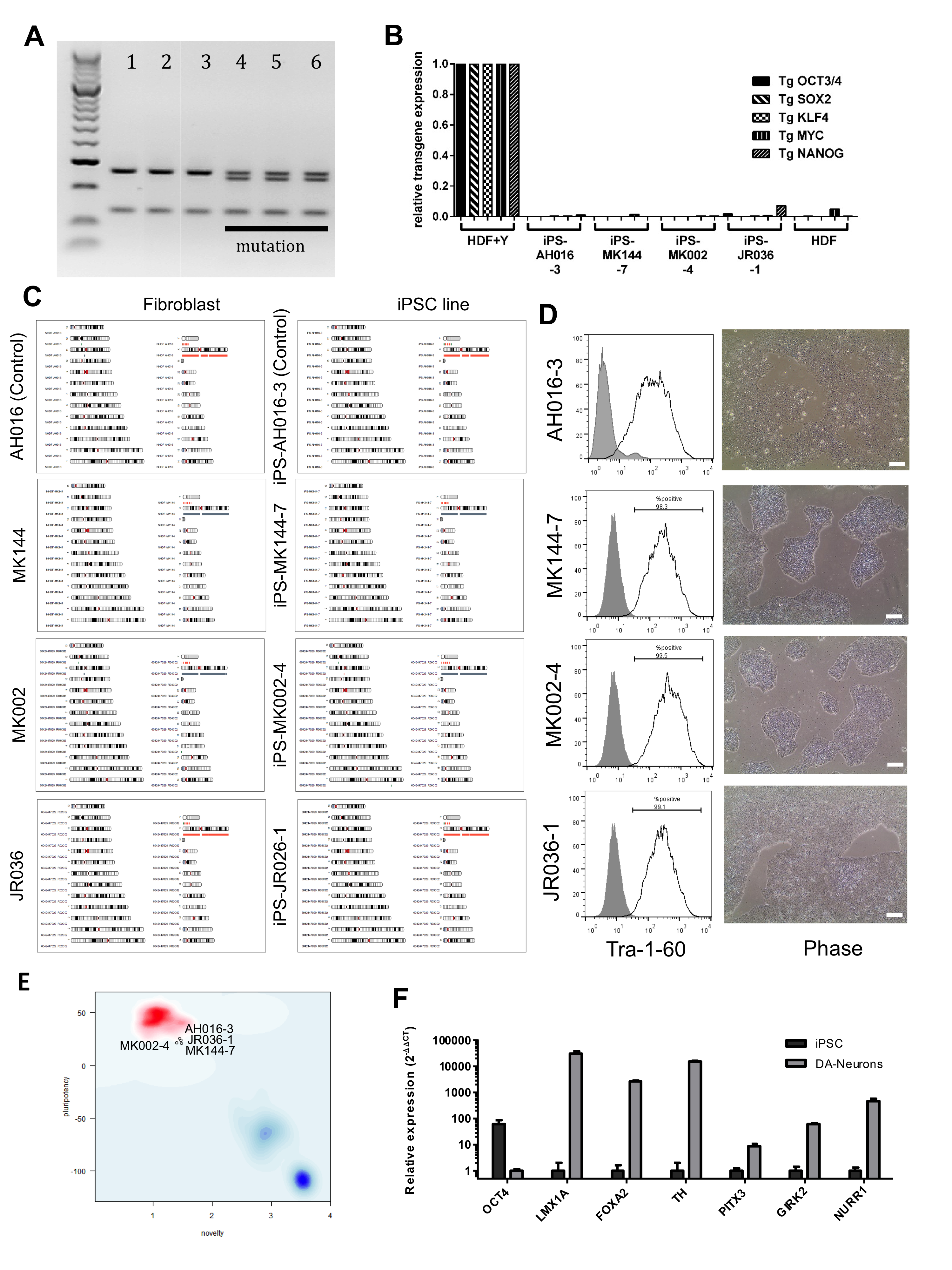
**

**Supplementary Figure1: Characterisation of iPSC and DaN lines**

(A) *G2019S* mutation screening on control (1,2 and 3) and *GBA* (4,5 and 6) samples.

(B) Transgene silencing in iPSC lines. qRT-PCR for each transgene, normalised to actin endogenous control, and expressed in comparison to the expression of transgenes from fibroblasts 5 days post-infection with the Yamanaka reprogramming retroviruses (HDF+Y). Uninfected fibroblasts (HDF) serve as a negative control.

(C) Genome integrity was assessed by Illumina Human CytoSNP-12v2.1 SNP array and karyograms produced using KaryoStudio software (Illumina). Amplifications (green), deletions (orange) and LOH regions (grey) are shown alongside the relevant chromosome (except that in females the X chromosomes are annotated with grey, and single-copy sex chromosomes are annotated orange).

(D) iPSC lines express the pluripotency protein Tra-1-60, as shown by FACs (grown feeder-free; open black plot represents antibody, filled grey plot represents isotype control), and show the expected iPSC colony morphology, with high nucleus to cytoplasm ratio by phase-contrast microscopy (right-hand panel, Scale bar = 100 µm). (E) PluriTest analysis of Illumina HT12v4 transcriptome array data shows the tested iPSC lines cluster together in the red cloud (correlating with pluripotency) and not with differentiated cells (blue clouds). Each circle represents one iPSC line.

(F) Representative quantitative real-time PCR (RT-PCR) demonstrating successful differentiation of control and PD *LRKK2-G2019S* iPSCs to dopaminergic (DA) neurons. Graph is mean ± SEM, N=3.

**Supplementary Figure 2: Number of live TH positive neurons collected and RIN numbers of RNA purified DaNs**

(A) table and (B) graph of the number of live TH+ cells collected for each sample. (C) RNA Integrity (RIN) analysis of control and LRKK2-G2019S sorted DaNs


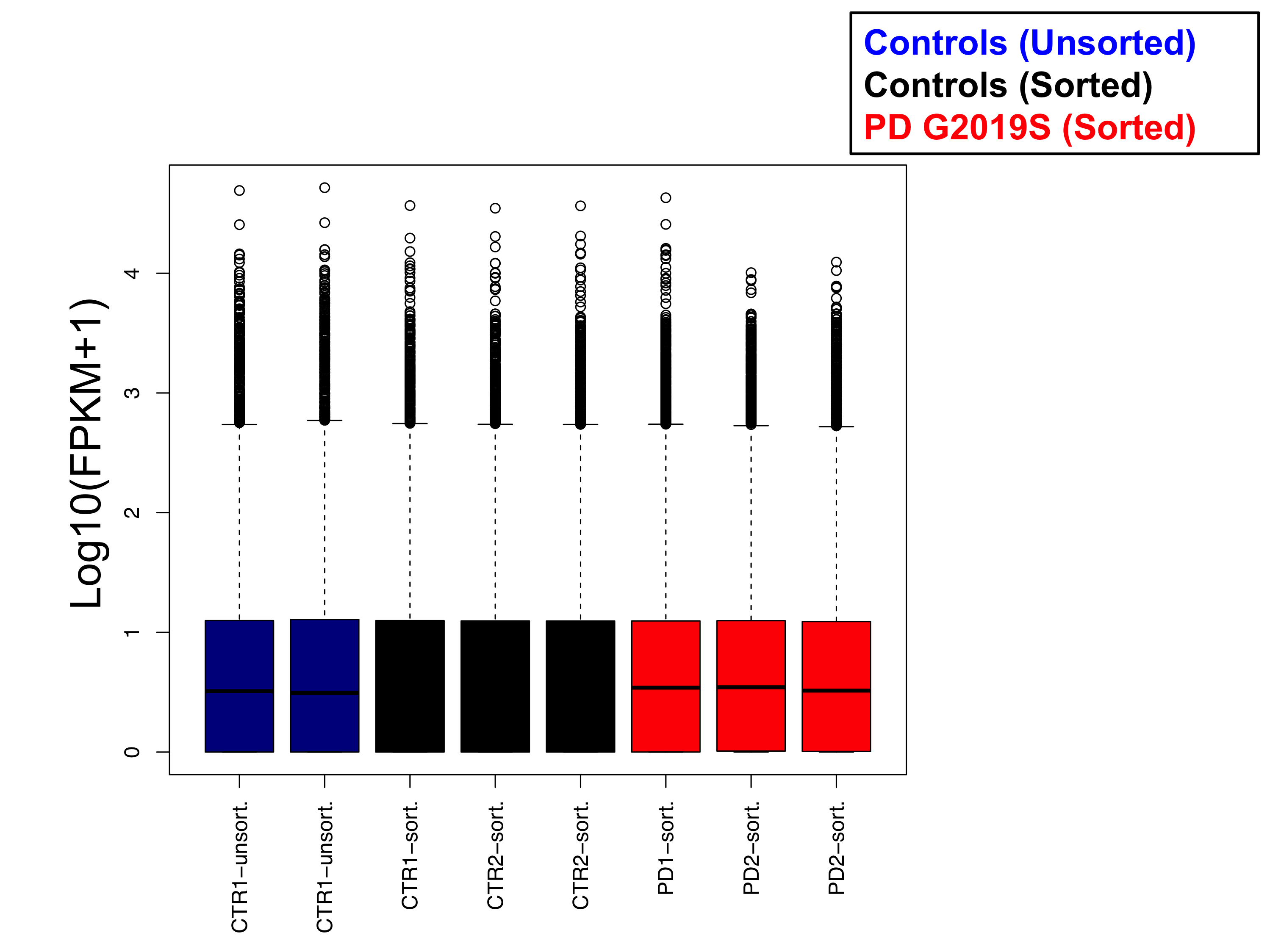


**Supplementary Figure 3: Distribution of expression level of 20158 protein-coding genes of eight iPSC derived dopaminergic midbrain neurons populations.**

The transcriptional profiles have been generated on six purified cells neurons populations coming from three PD patients carrying mutation *G2019-LRRK2* (red) and three controls (black) and on two non purified neurons population coming from two of three controls used to generated purified neurons populations (blue). The expression level measure is expressed in logarithm ten of reads per kilobase per million (RPKM) plus one. The two vertical dotted lines represent the 50nd (gray) and 75nd (black) percentiles of expression level measure.


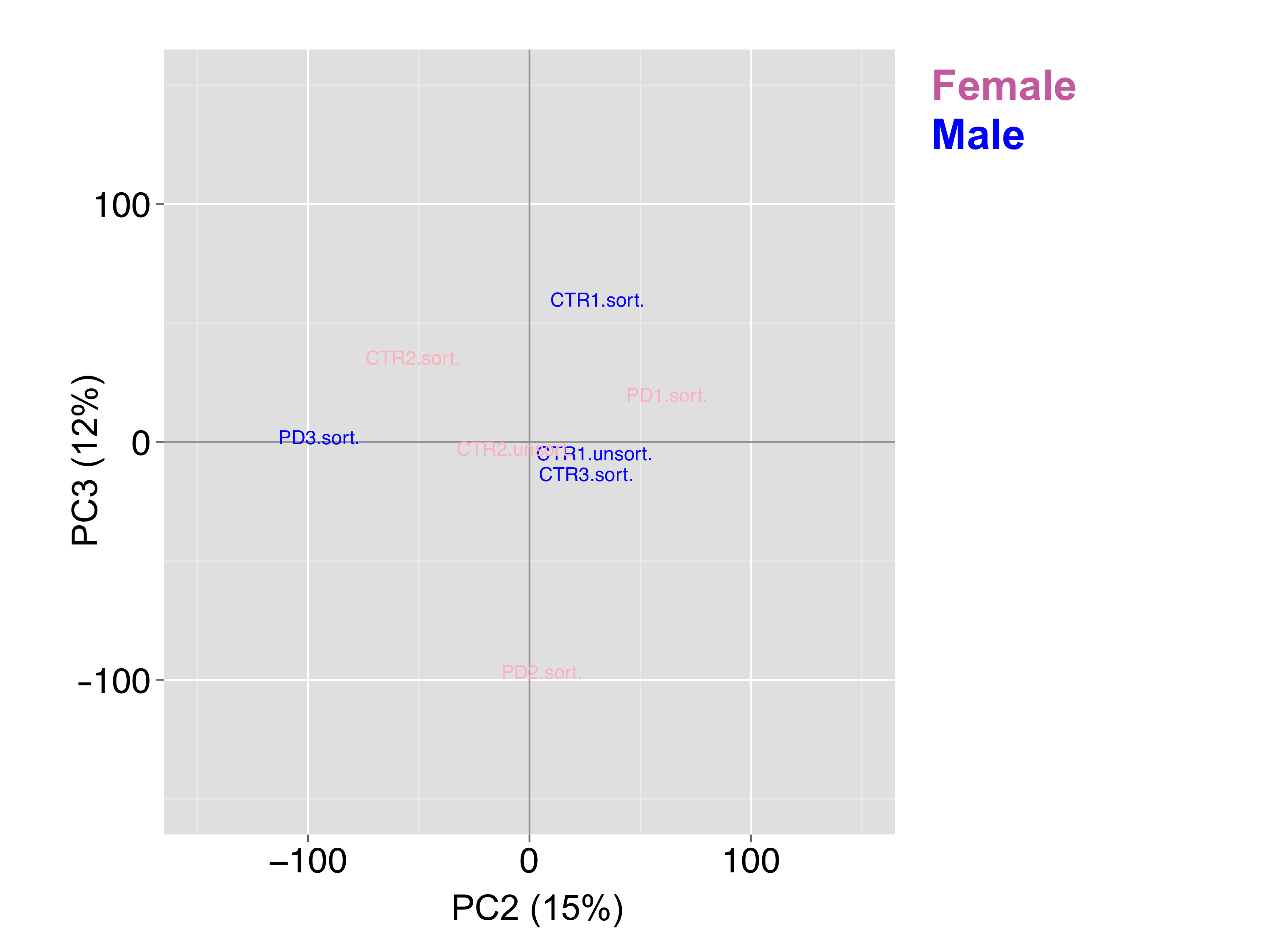


**Supplementary Figure 4: Gender effect on the transcriptional profiles**

Principal component analyses performed from FPKM values of 17170 of 20157 protein coding genes for which the variance was different zero. x and y axis represent the principal component 2 and 3 explaining 15% and 12% of variance respectively. The samples are annotated according to gender.


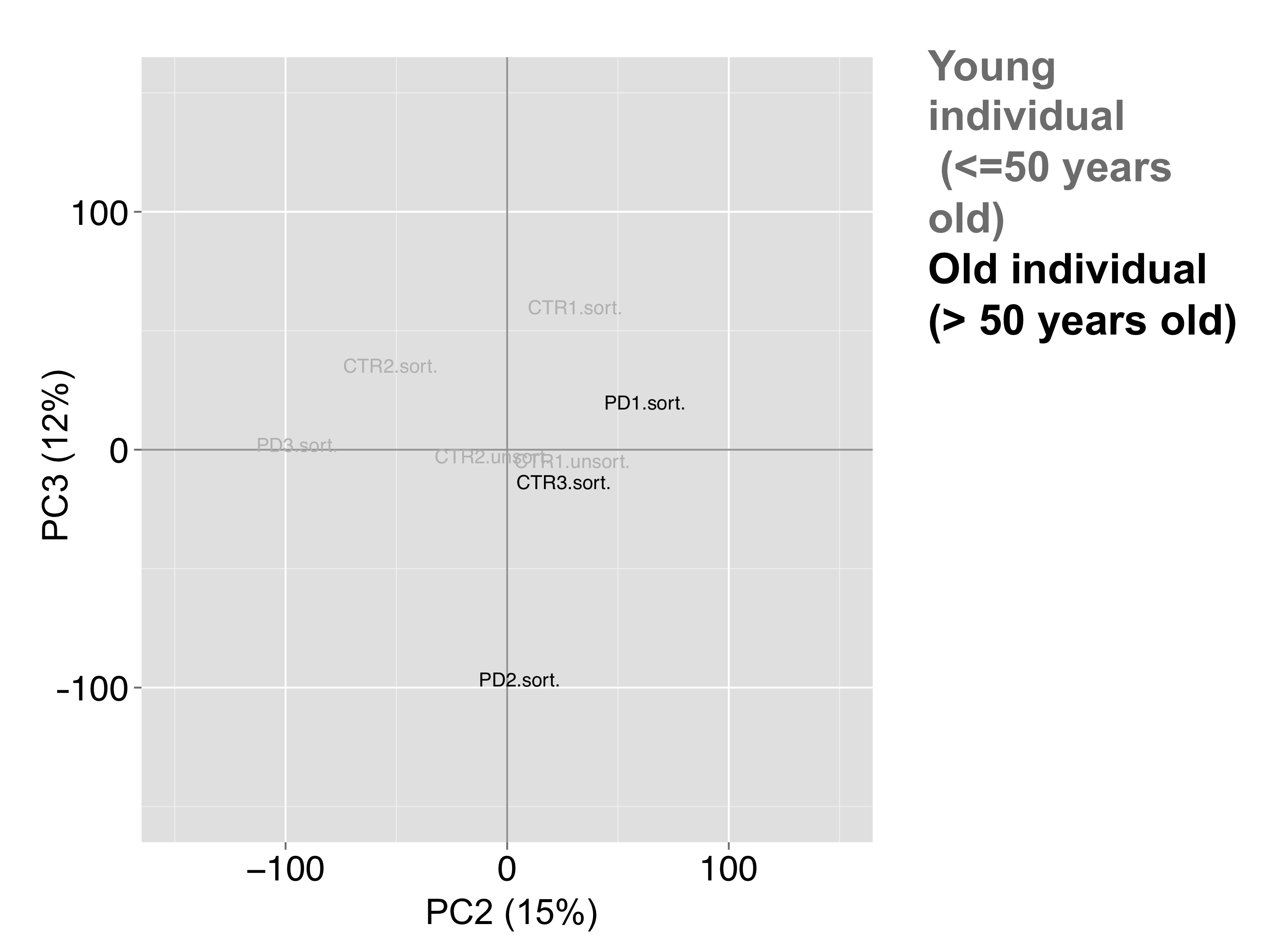


**Supplementary Figure 5: Age effect on the transcriptional profiles**

Principal component analyses performed from FPKM values of 17170 of 20157 protein coding genes for which the variance was different zero.

x and y axis represent the principal component 2 and 3 explaining 15% and 12% of variance respectively. The samples are annotated according to the relative age.


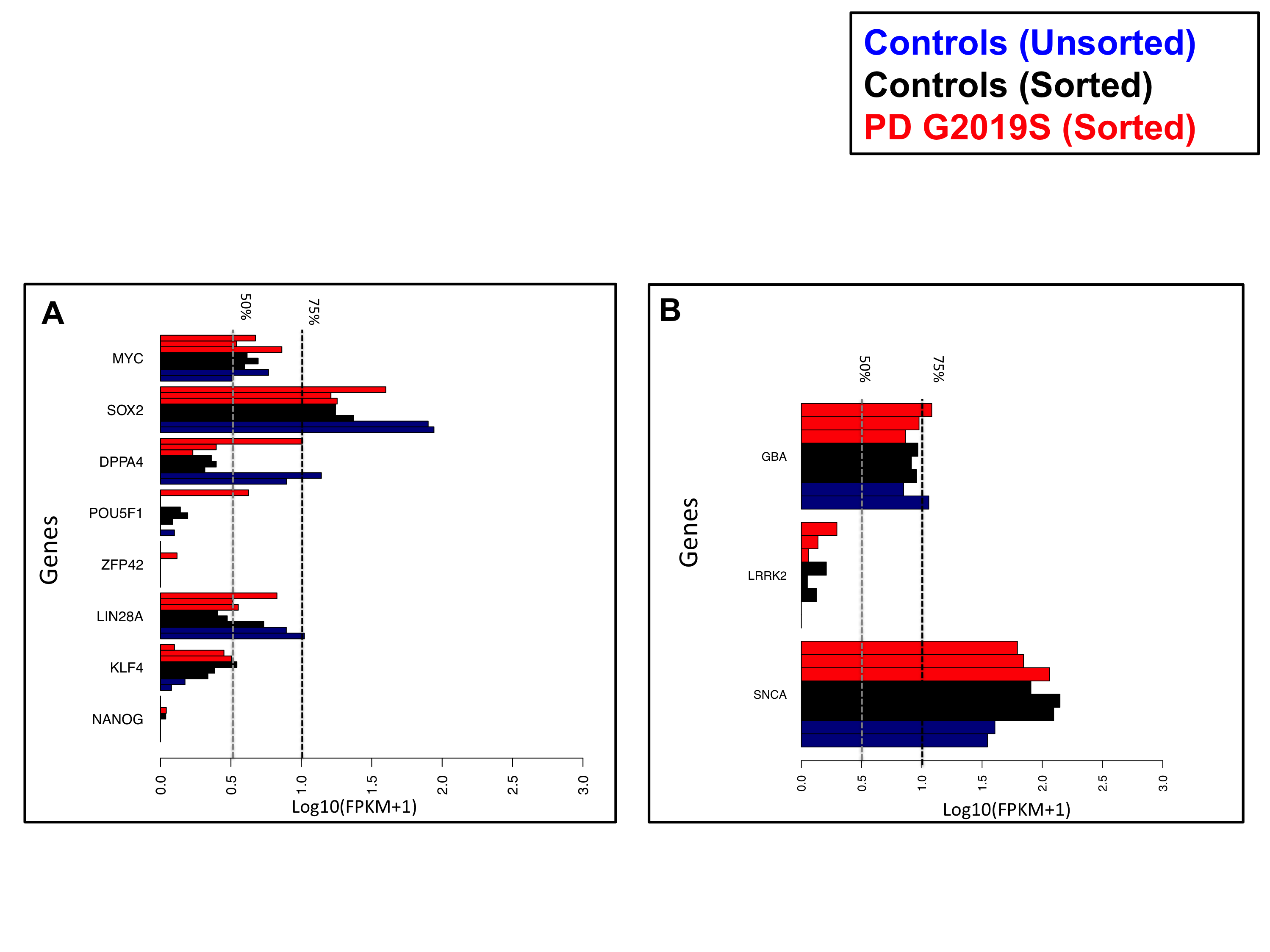


**Supplementary Figure 6: Expression level of eight pluripotency gene markers (A) and three Parkinson’s disease gene markers (B)**

The transcriptional profiles have been generated on six purified cells neurons populations coming from three PD patients carrying mutation *G2019-LRRK2* (red) and three controls (black) and on two non purified neurons population coming from two of three controls used to generated purified neurons populations (blue). The expression level measure is expressed in logarithm ten of reads per kilobase per million (RPKM) plus one. The two vertical dotted lines represent the 50nd (gray) and 75nd (black) percentiles of expression level measure.


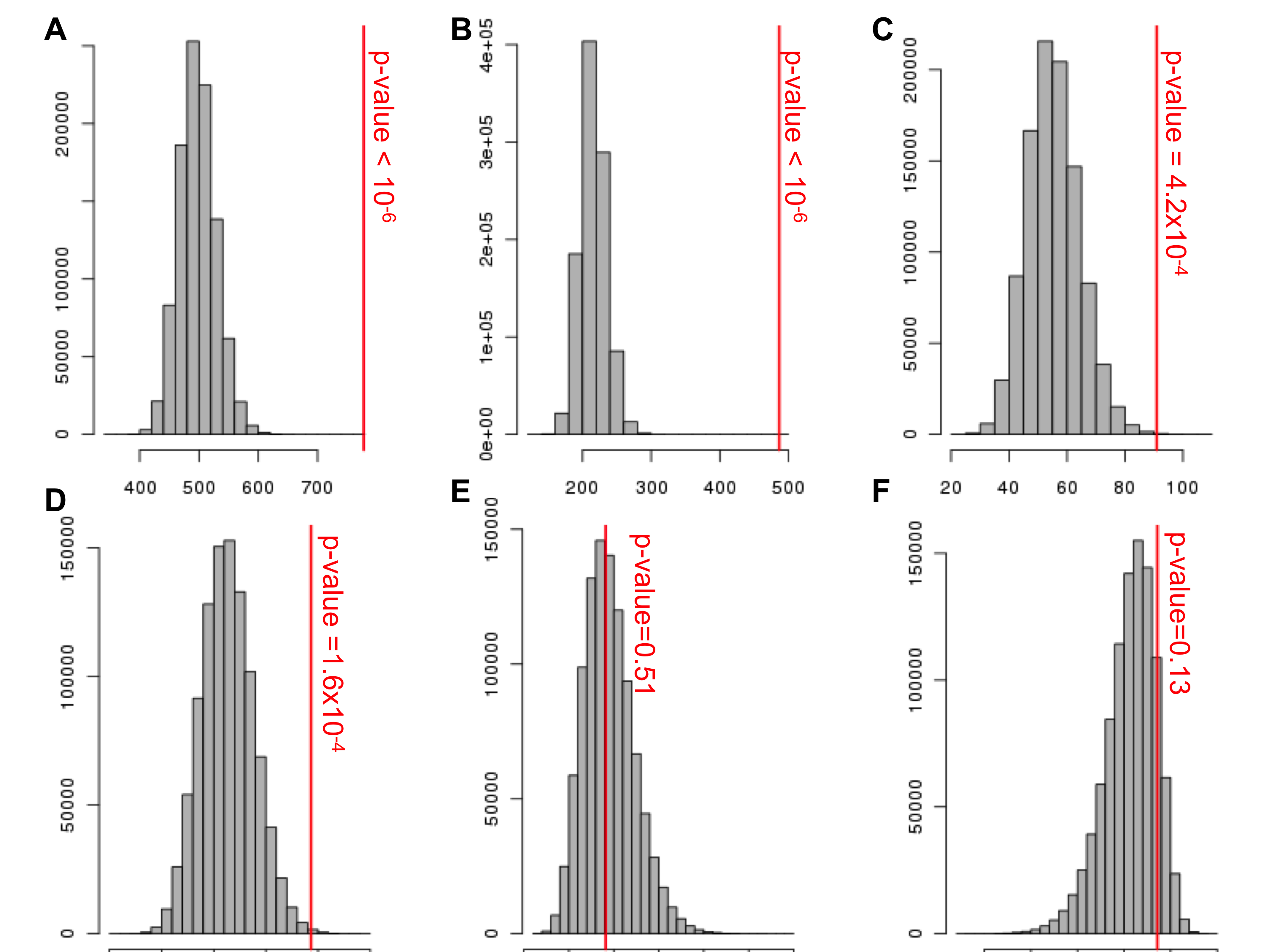


**Supplementary Figure 7: Clustering analyses within general Phenotypic Linkage Network of 168 DE genes**

The clustering analysis consist to compare the sum of weighted link within PLN (red line) for a gene set or between two gene set with random gene set or between two random genes set (gray histogram). The Figure A,B,C examine the clustering of 168 DE, 109 upregulated DE and 59 DE downregulated genes. The Figure D,F,G examine the clustering between up regulated genes, down regulated and between up and down regulated before and after permutation of up and down regulated status.


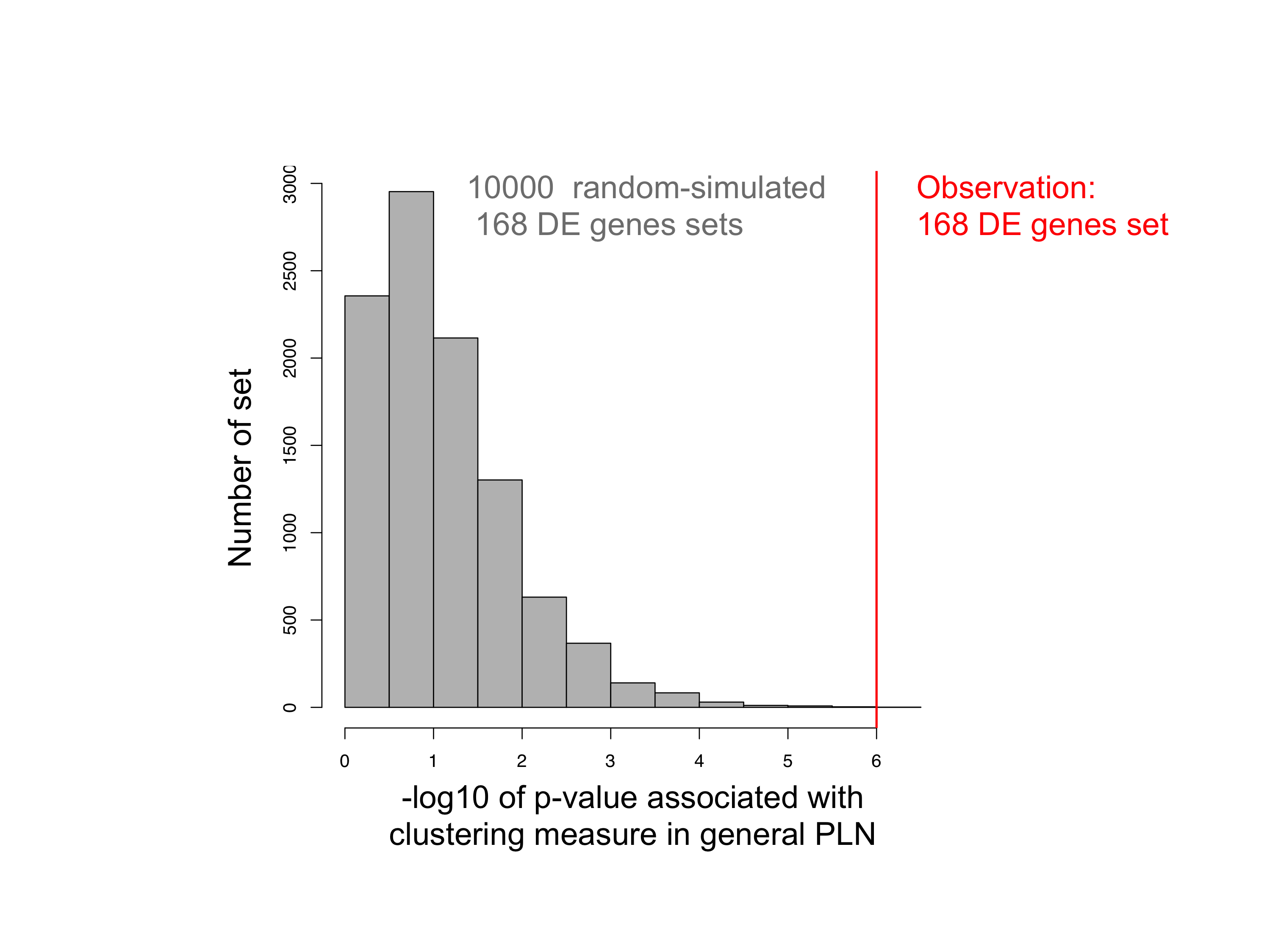


**Supplementary Figure 8: Comparison clustering analyses within general PLN of 168 DE genes with 10,000 simulated set of 168 DE.**

We generated random transcriptome profile for 10,000 genes set for 3 cases and 3 controls by using a binomial distribution where parameters x and y were estimated from RNA seq counts of our observations (see Methods). We then repeated the same differential expression analyses than for our transcriptional experiments and we performed clustering analyses by considering each time the 168 most DE genes.

The histogram represents the distribution of log10 of pval used to estimated the significant of measure of clustering of each genes set (sum of weighted links) within a general PLN.


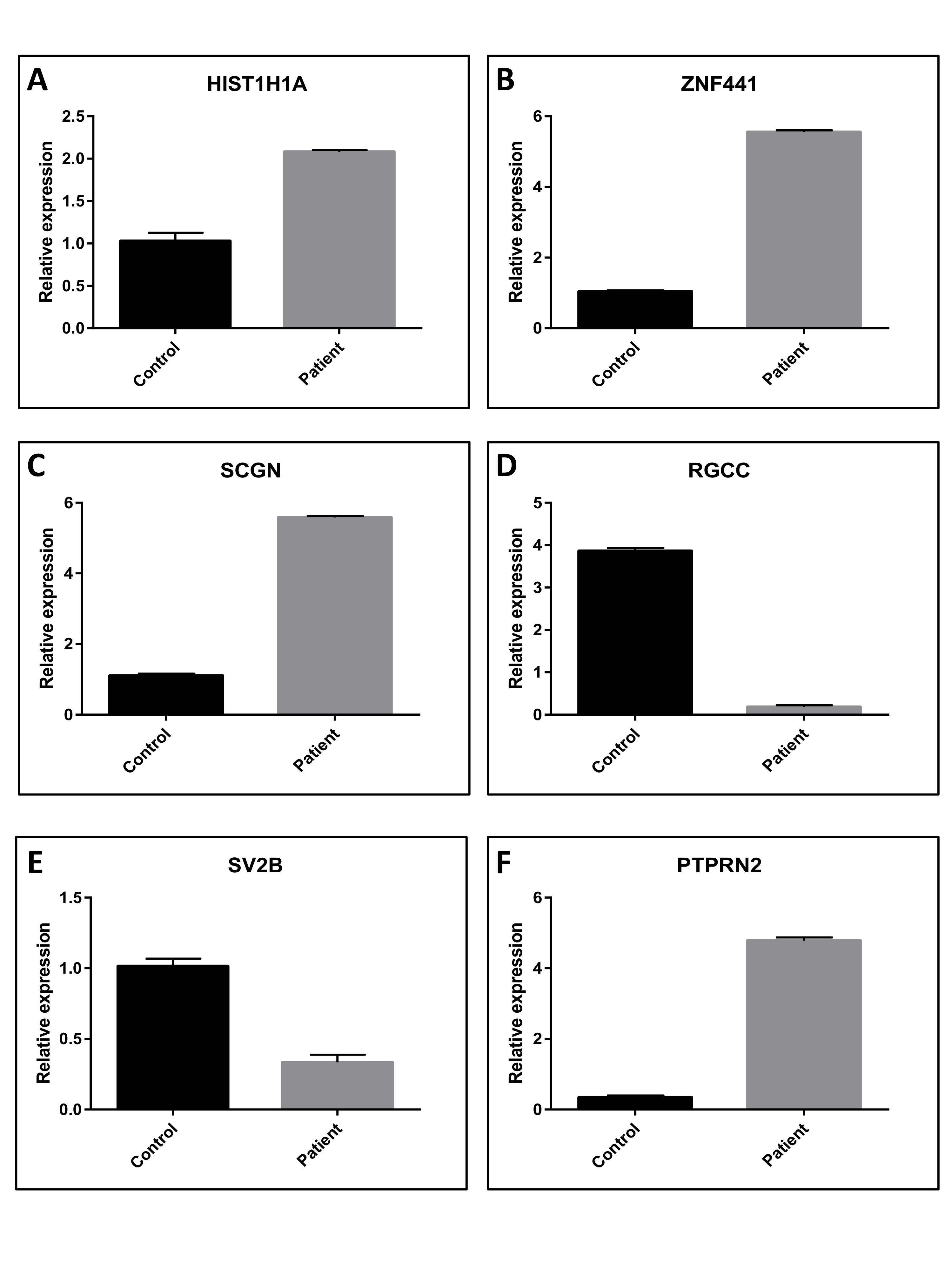


**Supplementary Figure 9:** qRT-PCR validation of top LRKK2 differentially expressed genes

The relative expression of six most differentially genes (A-F) identified by RNA-Seq (**Supplementary data 1**) were confirmed by qRT-PCR. The data represents both up and down regulated genes from three purified control and PD LRKK2-G2019S lines.

**
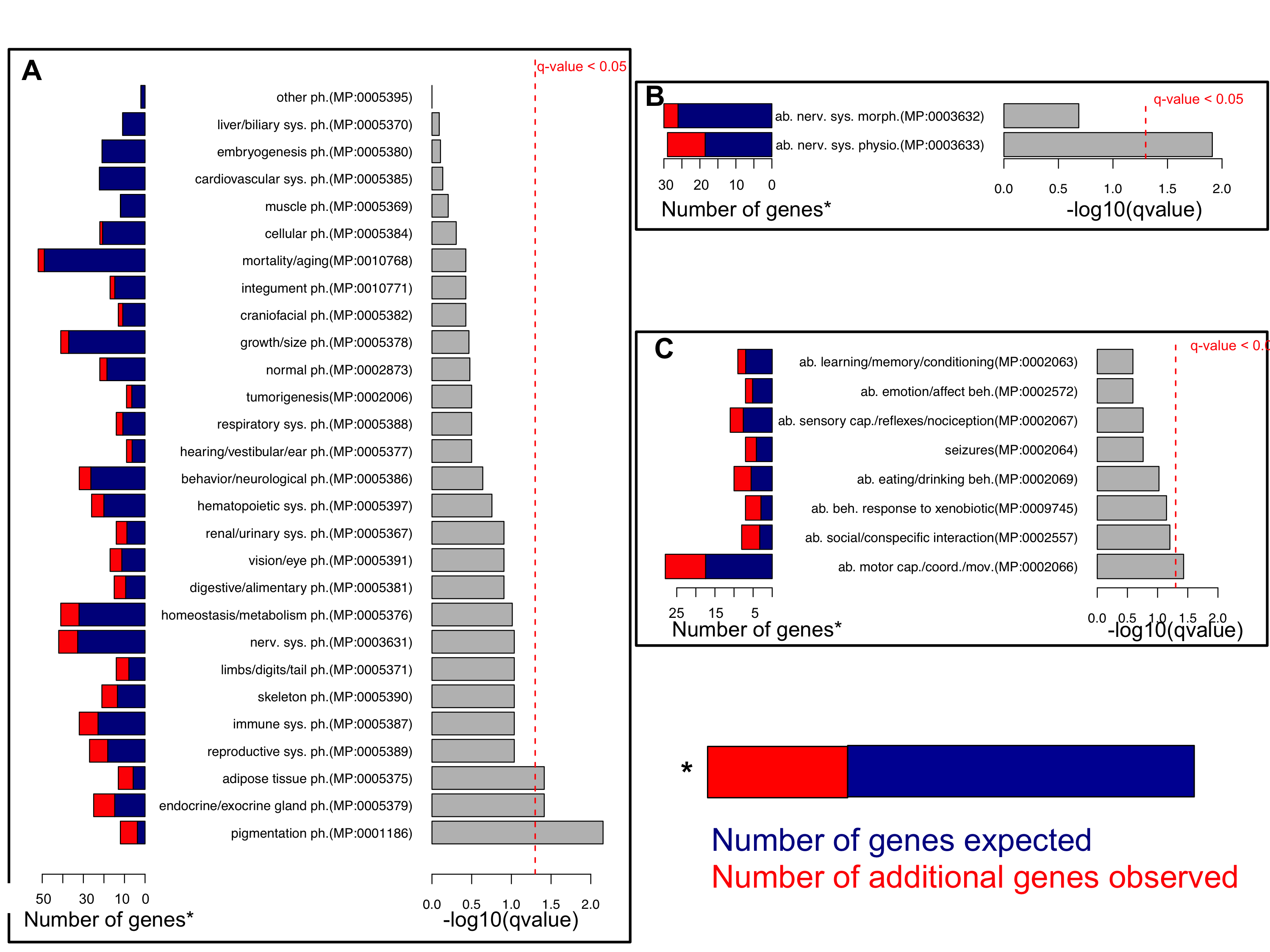
**

**Supplementary Figure 10: Phenotypes enriched following the disruption of the unique mouse orthologues of 168 DE genes.**

We considered 28/33 over-arching phenotypic categories (A), the children of nervous system phenotype (MP:0003631) (B) and behaviour neurological phenotype (MP:0005386) (C) For each phenotypic category tested, the right of part of each graph provide the -log of qvalue, while the left give the number of expected genes (blue) and the number of excess genes observed. Dotted line represents the significance threshold (FDR < 0.05).


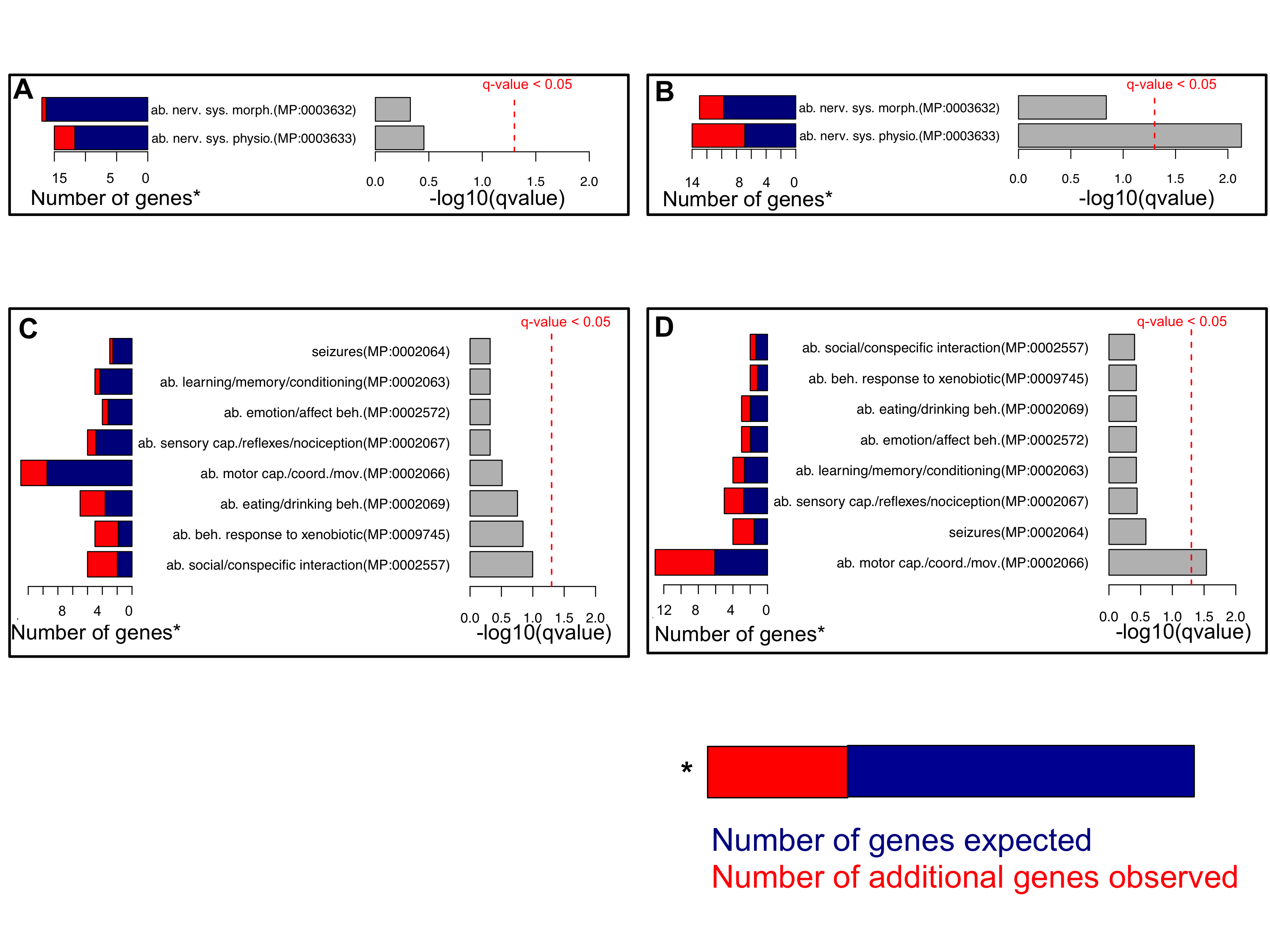


**Supplementary Figure 11: Phenotypes enriched following the disruption of the unique mouse orthologous of 109 up regulated genes (A,C) and 59 down regulated genes (B,D) in *LRRK2-G2019S* iPSC models.**

We considered the children of nervous system phenotype (MP:0003631) (A,B) and behavior neurological phenotype (MP:0005386) (C,D). For each phenotypic category tested, the right of part of each graph provide the -log of qvalue, while the left give the number of expected genes (blue)


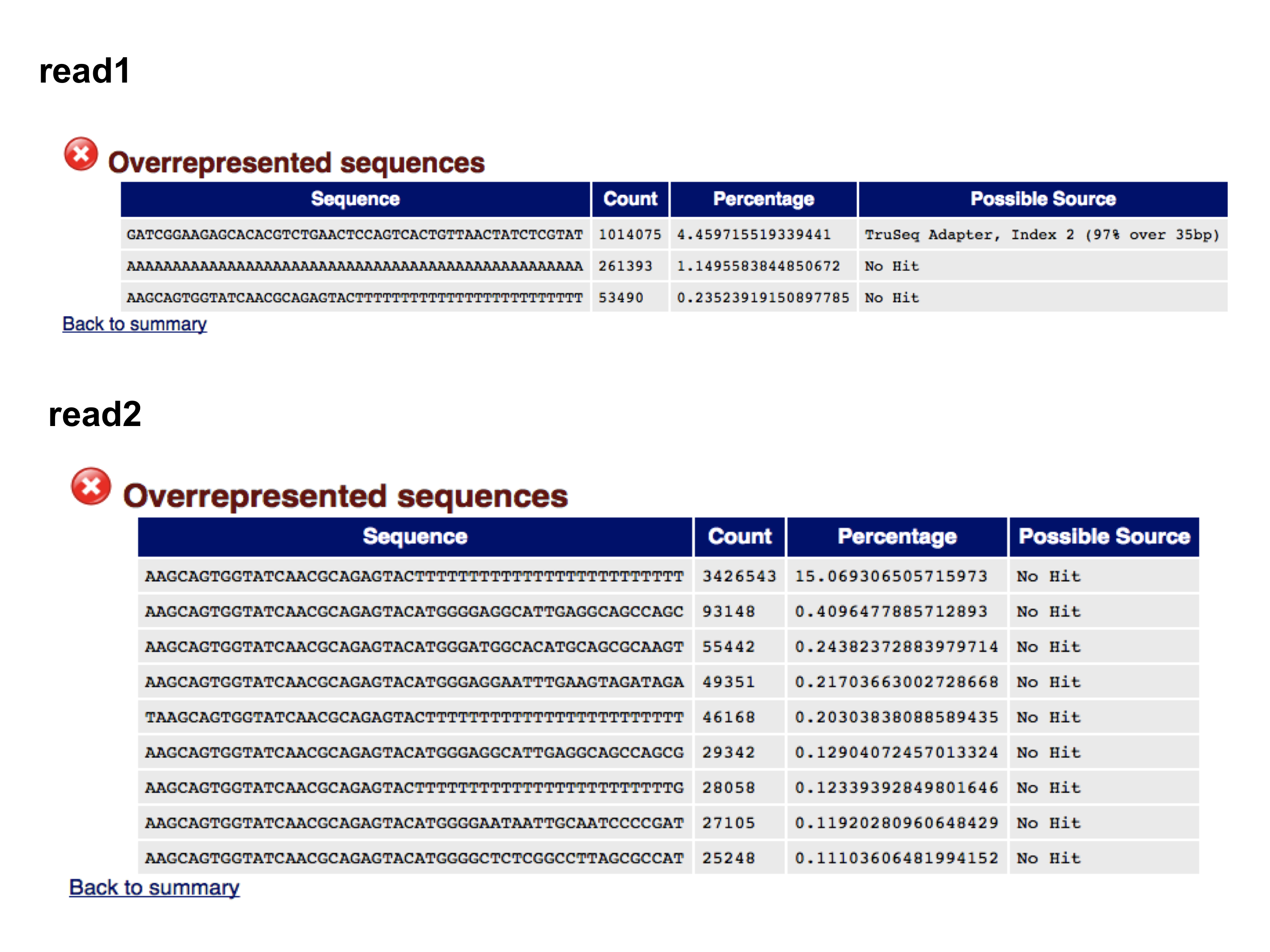


**Supplementary Figure 12: Illustration of adapter contamination for RNA sequence CTR3-sort. Line**


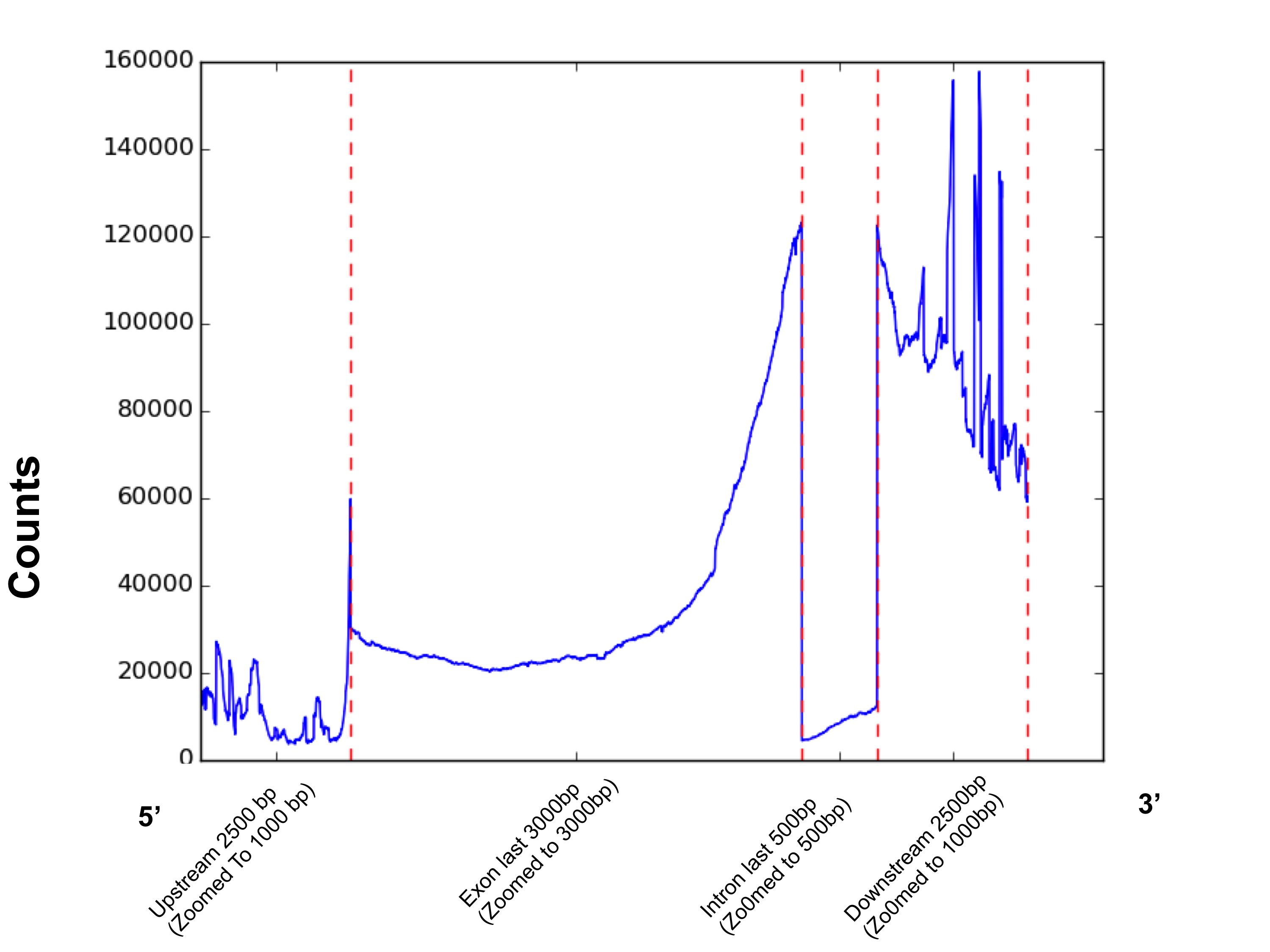


**Supplementary Figure 13: Illustration of 3'-5' bias with the meta-gene coverage profile of CTR3-sort. Line**

**Supplementary Table 1: Comparison of CMAP results (by name) for clioquinicol and rotenone for 168 DE genes observed in our experiment and 20 simulated set 168 DE genes as load signature**

|  |  | Clioquinol | |  | Rotenone | |
| --- | --- | --- | --- | --- | --- | --- |
| Observation | | enrichment | p |  | enrichment | p |
|  |  | -0.735 | 0.0027 |  | 0.75 | 0.0076 |
|  |  |  |  |  |  |  |
| simulations | 1 | -0.286 | 1 |  | -0.314 | 1 |
|  | 2 | -0.345 | 1 |  | 0.556 | 1 |
|  | 3 | -0.441 | 1 |  | -0.301 | 1 |
|  | 4 | 0.397 | 1 |  | 0.432 | 0.3343 |
|  | 5 | -0.462 | 1 |  | 0.298 | 1 |
|  | 6 | -0.416 | 1 |  | 0.392 | 1 |
|  | 7 | -0.46 | 1 |  | 0.59 | 1 |
|  | 8 | 0.395 | 1 |  | 0.39 | 0.46178 |
|  | 9 | 0.203 | 1 |  | 0.563 | 0.09998 |
|  | 10 | -0.377 | 1 |  | -0.335 | 1 |
|  | 11 | -0.61 | 1 |  | -0.428 | 0.34892 |
|  | 12 | -0.49 | 1 |  | 0.413 | 1 |
|  | 13 | 0.562 | 0.05173 |  | -0.386 | 0.48075 |
|  | 14 | -0.484 | 1 |  | 0.463 | 1 |
|  | 15 | -0.458 | 1 |  | 0.662 | 0.02982 |
|  | 16 | 0.309 | 1 |  | -0.417 | 0.38171 |
|  | 17 | -0.389 | 1 |  | 0.256 | 1 |
|  | 18 | 0.521 | 0.08729 |  | -0.398 | 1 |
|  | 19 | -0.554 | 1 |  | 0.483 | 0.21212 |
|  | 20 | 0.4 | 0.30948 |  | 0.578 | 0.08556 |

**Supplementary Table 2: Summary of quality control performed with FASTQC software (version 0.9.3)**

|  | **Basic Statistics** | **Per base sequence quality** | **Per sequence quality scores** | **Per base sequence content** | **Per base GC content** | **Per base N content** | **Sequence Length Distribution** | **Sequence Duplication Levels** | **Overrepresented sequences** | **Kmer Content** |
| --- | --- | --- | --- | --- | --- | --- | --- | --- | --- | --- |
| **Live-OX119_1_fastqc** | PASS | PASS | PASS | PASS | PASS | PASS | WARN | FAIL | PASS | WARN |
| **Live-OX119_2_fastqc** | PASS | PASS | PASS | FAIL | FAIL | PASS | WARN | FAIL | WARN | FAIL |
| **THplus_OX119_1_fastqc** | PASS | PASS | PASS | PASS | PASS | PASS | WARN | FAIL | FAIL | WARN |
| **THplus_OX119_2_fastqc** | PASS | PASS | PASS | FAIL | FAIL | PASS | WARN | FAIL | WARN | FAIL |
| **Live-NHDF1_1_fastqc** | PASS | PASS | PASS | PASS | PASS | PASS | WARN | FAIL | FAIL | WARN |
| **Live-NHDF1_2_fastqc** | PASS | PASS | PASS | FAIL | FAIL | PASS | WARN | FAIL | WARN | FAIL |
| **THplus-NHDF1_1_fastqc** | PASS | PASS | PASS | PASS | PASS | PASS | WARN | FAIL | PASS | WARN |
| **THplus-NHDF1_2_fastqc** | PASS | PASS | PASS | FAIL | FAIL | PASS | WARN | FAIL | WARN | FAIL |
| **THplus-AH016-1_fastqc** | PASS | PASS | PASS | PASS | PASS | PASS | WARN | FAIL | PASS | WARN |
| **THplus-AH016-2_fastqc** | PASS | PASS | PASS | FAIL | FAIL | PASS | WARN | FAIL | WARN | FAIL |
| **THplus-J036-1_fastqc** | PASS | PASS | PASS | PASS | PASS | PASS | WARN | FAIL | PASS | WARN |
| **THplus-J036-2_fastqc** | PASS | PASS | PASS | FAIL | FAIL | PASS | WARN | FAIL | WARN | FAIL |
| **THplus-MK002-1_fastqc** | PASS | PASS | PASS | PASS | PASS | PASS | WARN | FAIL | PASS | WARN |
| **THplus-MK002-2_fastqc** | PASS | PASS | PASS | FAIL | FAIL | PASS | WARN | FAIL | WARN | FAIL |
| **THplus-MK144-1_fastqc** | PASS | PASS | PASS | PASS | PASS | PASS | WARN | FAIL | PASS | WARN |
| **THplus-MK144-2_fastqc** | PASS | PASS | PASS | FAIL | FAIL | PASS | WARN | FAIL | WARN | FAIL |

**Supplementary Table 3: Summary of read alignments performed with STAR**

| Category | Counts* | Percent of* |  |
| --- | --- | --- | --- |
| alignments_total | 98573406.5 | alignments_total | 100 |
| alignments_mapped | 75496260 | alignments_total | 76.56 |
| alignments_unmapped | 23077146.5 | alignments_total | 23.44 |
| alignments_qc_fail | 0 | alignments_mapped | 0 |
| alignments_mate_unmapped | 23077146.5 | alignments_mapped | 30.64 |
| alignments_reverse | 37748130 | alignments_mapped | 50 |
| alignments_mate_reverse | 37748130 | alignments_mapped | 50 |
| alignments_proper_pair | 75496260 | alignments_mapped | 100 |
| alignments_read1 | 49286703.25 | alignments_mapped | 65.32 |
| alignments_paired | 98573406.5 | alignments_mapped | 130.64 |
| alignments_duplicate | 0 | alignments_mapped | 0 |
| alignments_read2 | 49286703.25 | alignments_mapped | 65.32 |
| alignments_secondary | 10957782.25 | alignments_mapped | 14.5475 |
| alignments_rna | 0 | alignments_mapped | 0 |
| alignments_no_rna | 0 | alignments_mapped | 0 |
| alignments_filtered | 0 | alignments_mapped | 0 |
| reads_total | 87615624.25 | reads_total | 100 |
| reads_mapped | 64538477.75 | reads_total | 73.6175 |
| reads_unmapped | 23077146.5 | reads_total | 26.3825 |
| reads_missing | 0 | reads_total | 0 |
| reads_unique | 59647040.75 | reads_mapped | 92.41875 |
| reads_norna_unique | 59647040.75 | reads_mapped | -545.17125 |
| pairs_total | 43807812.13 | pairs_total | 100 |
| pairs_mapped | 37748130 | pairs_total | 86.145 |

* Mean estimated on 8 libraries

**Supplementary Table 4: Gene pairs coverage for each different individual dataset and the final PLN**

| Dataset | Number of gene pairs after rescale |
| --- | --- |
| GO annotations biological process | 5807378 |
| GO annotations cellular locations | 235529 |
| GO annotations molecular process | 2049681 |
| Text literature | 423076 |
| Co-expression (8 microarray dataset used in Honti *et al.* (PMID: 25166029) | 6956398 |
| Kegg & reactome | 1434285 |
| Protein-Protein Interactions | 124352 |
| Co-expression based on RNAseq experiments (Brainspan) PMID: 23193282 | 151636 |
| Co-expression based on RNAseq experiments (GTEx) PMID: 1262110 | 6133742 |
| Interprotein domains | 1837482 |
| Totat number of links | 20827920 |

**Supplementary Note1: Generation of human iPSC**

All iPSC lines used in this study were derived from skin biopsies and reprogrammed using the same methodology and in the same laboratory. Two lines have been published previously iPS-OX1-19 (1) and iPS-NHDF-1(1, 2). 4mm skin punch biopsies were cut into several small pieces and cultured under glass coverslips in ADMEM (Invitrogen) supplemented with USDA-approved foetal bovine serum (10%; Sigma) and penicillin/streptomycin (1%) in a humidified incubator (37 °C, 5% CO_2_). 50,000 fibroblasts were reprogrammed at passage 3-5, using retroviruses carrying individual reprogramming genes (Addgene pMXs plasmids -17220: pMXs-hc-MYC, 17219: pMXs-hKLF4, 17218: pMXs-hSOX2, 17217: pMXs-hOCT3/4, 13354: pMXs-Nanog, packaged using the Plat-GP retroviral packaging cell line (containing retroviral gag and pol genes) with VSV-G envelope)(3-5) . Virus ratios were O,S,K,M,N:3,1,1,1,1 on days 0 and 1 with 5 µg/ml polybrene and spinoculation (1200 g for 45 minutes at 16^o^C). Fibroblasts were transferred onto mitotically-inactivated ‘Pathology Oxford’ (6) outbred mouse embryonic feeder cells (MEF) on 0.1% gelatin coated plates (Sigma) on day 4, and from day 5 onwards were cultured in standard KnockOut serum replacement medium (Knock-out DMEM (Invitrogen), KO-Serum Replacement (20% Invitrogen), Glutamax-I (2 mM Invitrogen), non-essential amino acids (1%, Invitrogen), penicillin (100 U/mL Invitrogen), streptomycin (100 µg/mL Invitrogen), 2-ME (55 µM Invitrogen) and bFGF (10 ng/mL R&D)) (with 50 µg/ml ascorbic acid and 0.5 µM Valproic acid (both from Sigma) to enhance reprogramming efficiency), replacing 50% medium on alternate days (MEF-conditioned medium from day 10). Colonies displaying iPSC morphology were picked on day ~28 and passaged routinely on MEFs by manual dissection. iPSC lines were adapted to feeder-free culture conditions on Matrigel-coated plates (BD Matrigel hESC-qualified Matrix) in mTeSR™1 (StemCell Technologies), using 0.5 mM EDTA in PBS to dissociate cells to small clumps(7). For some applications, enzymatic dissociation (TryplE Express, Gibco) supplemented with Rock inhibitor Y27632 (10 μM; Calbiochem) on the day of passage was used. – in this case, the number of feeder-free passages was always kept to an absolute minimum to reduce the likelihood of genetic change. Cells were frozen in SNP-QCed batches of at least 30 vials (within a narrow window of passage numbers, ~p15-30), from which cells would be thawed for each experiment, to ensure consistency across experiments.

**Supplementary Note2: Characterisation of iPSC**

Expression of the key pluripotency marker TRA-1-60 was assessed by fluorescence activated cell sorting (FACs), with appropriate isotype control at the same concentration, from the same supplier (clone B119983, IgM-488, Biolegend). Fixation was for 10 minutes in 2% paraformaldehyde in PBS (Alfa Aesar). Fluorescence was measured using a FACS Calibur (Becton Dickinson), data was analysed using FlowJo software.

qRT-PCR for assessing the degree of silencing of retroviral transgene sequences used primer sequences published by Takahashi et al.(5) , substituting primer pMXs-AS3200 with pMXs-AS3200v2 (TTA TCG TCG ACC ACT GTG CTG GCG) and for mNanog, the forward primer GCT CCA TAA CTT CGG GGA GG was used. RNA was reverse transcribed using a RetroScript kit (Ambion) (2ug RNA in 20 µl). 2 µl of 1:10 dilution of cDNA was used in 25 µl qRT-PCR reaction, on an Applied Biosystems StepOne Plus Real Time PCR machine, with StepOne software, using Applied Biosystems 2xSYBR green PCR mix + ROX and 60°C anneal. Target gene transcript levels were compared to actin B control (actin B primers, Eurogentec), and subsequently to fibroblasts harvested 5 days after infection with the reprogramming vectors.

Analysis of pluripotency was performed on RNA extracted from iPSC lines using the Illumina HT12v4 transcriptome array. The image data files were uploaded to www.pluritest.org and scored for pluripotency, as previously described(8). Pluripotent cell lines cluster in the top left quadrant of the graph, indicating high pluripotency score and low novelty score.

Genome integrity and cell tracking was assessed using Illumina Human CytoSNP-12v2.1 beadchip array (~300,000 markers) and analysis with KaryoStudio and GenomeStudio software (Illumina), comparing the SNP profiles of the iPSC lines to the parental fibroblasts.

**Supplementary Note 3: Differential expression analysis with limma R bioconductor package**

# Version info: R 3.2.3, Biobase 2.30.0, GEOquery 2.36.0, limma 3.26.8

library(Biobase)
library(GEOquery)
library(limma)

# load series and platform data from GEO

gset <- getGEO("GSE43364", GSEMatrix =TRUE)
if (length(gset) > 1) idx <- grep("GPL10558", attr(gset, "names")) else idx <- 1
gset <- gset[[idx]]

# make proper column names to match toptable 
fvarLabels(gset) <- make.names(fvarLabels(gset))

# group names for all samples
sml <- c("G1","G0","G1","G0","G1","G0","X","X");

# eliminate samples marked as "X"
sel <- which(sml != "X")
sml <- sml[sel]
gset <- gset[ ,sel]

# log2 transform
ex <- exprs(gset)
qx <- as.numeric(quantile(ex, c(0., 0.25, 0.5, 0.75, 0.99, 1.0), na.rm=T))
LogC <- (qx[5] > 100) ||
          (qx[6]-qx[1] > 50 && qx[2] > 0) ||
          (qx[2] > 0 && qx[2] < 1 && qx[4] > 1 && qx[4] < 2)
if (LogC) { ex[which(ex <= 0)] <- NaN
  exprs(gset) <- log2(ex) }

# set up the data and proceed with analysis
fl <- as.factor(sml)
gset$description <- fl
design <- model.matrix(~ description + 0, gset)
colnames(design) <- levels(fl)
fit <- lmFit(gset, design)
cont.matrix <- makeContrasts(G1-G0, levels=design)
fit2 <- contrasts.fit(fit, cont.matrix)
fit2 <- eBayes(fit2, 0.01)
tT <- topTable(fit2, adjust="fdr", [sort.by](http://sort.by/)="B",number=100000000)

# load NCBI platform annotation
gpl <- annotation(gset)
platf <- getGEO(gpl, AnnotGPL=TRUE)
ncbifd <- data.frame(attr(dataTable(platf), "table"))

# replace original platform annotation
tT <- tT[setdiff(colnames(tT), setdiff(fvarLabels(gset), "ID"))]
tT <- merge(tT, ncbifd, by="ID")
tT <- tT[order(tT$P.Value), ]  # restore correct order

tT <- subset(tT, select=c("ID","adj.P.Val","P.Value","AveExpr","t","B","logFC","Gene.symbol","Gene.title"))
write.table(tT, file=stdout(), row.names=F, sep="\t")

**References**

1 van Wilgenburg, B., Browne, C., Vowles, J. and Cowley, S.A. (2013) Efficient, long term production of monocyte-derived macrophages from human pluripotent stem cells under partly-defined and fully-defined conditions. *PLoS One*, **8**, e71098.

2 Hartfield, E.M., Yamasaki-Mann, M., Ribeiro Fernandes, H.J., Vowles, J., James, W.S., Cowley, S.A. and Wade-Martins, R. (2014) Physiological characterisation of human iPS-derived dopaminergic neurons. *PLoS One*, **9**, e87388.

3 Kitamura, T., Koshino, Y., Shibata, F., Oki, T., Nakajima, H., Nosaka, T. and Kumagai, H. (2003) Retrovirus-mediated gene transfer and expression cloning: powerful tools in functional genomics. *Exp Hematol*, **31**, 1007-1014.

4 Takahashi, K. and Yamanaka, S. (2006) Induction of pluripotent stem cells from mouse embryonic and adult fibroblast cultures by defined factors. *Cell*, **126**, 663-676.

5 Takahashi, K., Tanabe, K., Ohnuki, M., Narita, M., Ichisaka, T., Tomoda, K. and Yamanaka, S. (2007) Induction of pluripotent stem cells from adult human fibroblasts by defined factors. *Cell*, **131**, 861-872.

6 Gardner, R.L. (1982) Investigation of cell lineage and differentiation in the extraembryonic endoderm of the mouse embryo. *J Embryol Exp Morphol*, **68**, 175-198.

7 Beers, J., Gulbranson, D.R., George, N., Siniscalchi, L.I., Jones, J., Thomson, J.A. and Chen, G. (2012) Passaging and colony expansion of human pluripotent stem cells by enzyme-free dissociation in chemically defined culture conditions. *Nat Protoc*, **7**, 2029-2040.

8 Muller, F.J., Schuldt, B.M., Williams, R., Mason, D., Altun, G., Papapetrou, E.P., Danner, S., Goldmann, J.E., Herbst, A., Schmidt, N.O. *et al.* (2011) A bioinformatic assay for pluripotency in human cells. *Nat Methods*, **8**, 315-317.
